# Tailoring the Photophysical Properties of a Homoleptic Iron(II) Tetra *N*-Heterocyclic Carbene Complex by Attaching an Imidazolium Group to the (C^∧^N^∧^C) Pincer Ligand—A Comparative Study

**DOI:** 10.1021/acs.inorgchem.3c02890

**Published:** 2024-02-01

**Authors:** Om Prakash, Linnea Lindh, Arvind Kumar Gupta, Yen Tran Hoang Hai, Nidhi Kaul, Pavel Chábera, Fredrik Lindgren, Tore Ericsson, Lennart Häggström, Daniel Strand, Arkady Yartsev, Reiner Lomoth, Petter Persson, Kenneth Wärnmark

**Affiliations:** † Centre for Analysis and Synthesis, Department of Chemistry, Lund University, Box 124, Lund SE-22100, Sweden; ‡ Chemical Physics Division, Department of Chemistry, Lund University, Box 124, Lund SE-22100, Sweden; § Theoretical Chemistry Division, Department of Chemistry, Lund University, Box 124, Lund SE-22100, Sweden; ∥ Department of Chemistry—Ångström Laboratory, Uppsala University, Box 523, Uppsala SE-751 20, Sweden; ⊥ Department of Physics—Ångström Laboratory, Uppsala University, Box 523, Uppsala SE-751 20, Sweden

## Abstract

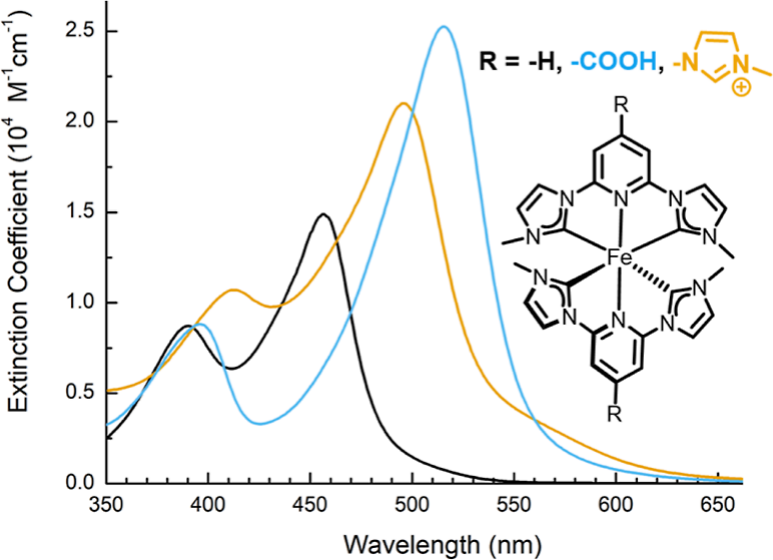

We here report the synthesis of the homoleptic iron(II) *N*-heterocyclic carbene (NHC) complex [Fe(miHpbmi)_2_](PF_6_)_4_ (miHpbmi = 4-((3-methyl-1*H*-imidazolium-1-yl)pyridine-2,6-diyl)bis(3-methylimidazol-2-ylidene)) and its electrochemical and photophysical properties. The introduction of the π-electron-withdrawing 3-methyl-1*H*-imidazol-3-ium-1-yl group into the NHC ligand framework resulted in stabilization of the metal-to-ligand charge transfer (MLCT) state and destabilization of the metal-centered (MC) states. This resulted in an improved excited-state lifetime of 16 ps compared to the 9 ps for the unsubstituted parent compound [Fe(pbmi)_2_](PF_6_)_2_ (pbmi = (pyridine-2,6-diyl)bis(3-methylimidazol-2-ylidene)) as well as a stronger MLCT absorption band extending more toward the red spectral region. However, compared to the carboxylic acid derivative [Fe(cpbmi)_2_](PF_6_)_2_ (cpbmi = 1,1′-(4-carboxypyridine-2,6-diyl)bis(3-methylimidazol-2-ylidene)), the excited-state lifetime of [Fe(miHpbmi)_2_](PF_6_)_4_ is the same, but both the extinction and the red shift are more pronounced for the former. Hence, this makes [Fe(miHpbmi)_2_](PF_6_)_4_ a promising pH-insensitive analogue of [Fe(cpbmi)_2_](PF_6_)_2_. Finally, the excited-state dynamics of the title compound [Fe(miHpbmi)_2_](PF_6_)_4_ was investigated in solvents with different viscosities, however, showing very little dependency of the depopulation of the excited states on the properties of the solvent used.

## Abstract 2

A pH-insensitive alternative to carboxylic acid substituents: electron-withdrawing methylimidazolium groups stabilize the MLCT state and destabilize the MC states of an iron(II) *N*-heterocyclic carbene complex and result in improved excited-state lifetime and stronger and red-shifted MLCT absorption compared to the unsubstituted parent compound.

## Introduction

First-row transition-metal complexes have the potential to replace precious and expensive second- and third-row transition-metal complexes, such as ruthenium and iridium, as efficient photosensitizers and photocatalysts.^1−3^ Some remarkable progress toward this end has been achieved using complexes based on chromium, manganese, and copper, demonstrating excited-state lifetimes up to the ns-range.^2,4,5^ Earth-abundant transition-metal complexes have been demonstrated as photosensitizers in dye-sensitized solar cells (DSSCs),^6,7^ in hydrogen-evolution reactions,^8^ as photocatalysts in photoredox catalysis,^9,10^ and as luminophores in OLEDs.^11,12^ Particularly interesting would be the employment of iron in metal complexes for such applications due to their abundance, low cost, and nontoxicity.^13−15^ Iron complexes as analogues of the archetypical ruthenium polypyridyl photosensitizers^16^ have therefore received large research interest.^17−24^

The problem with the traditional iron polypyridyl complexes for photophysical and photochemical applications using visible light has been their ultrafast deactivation of the photoactive charge-transfer (CT) states via energetically low-lying metal-centered (MC) states. This happens due to the weak ligand field splitting of first-row transition metals^18^ in combination with the pyridine ligands. Arguably, the best approach to this end has been achieved by the introduction of strongly σ-donating ligands that destabilize the MC states in an octahedral ligand field. Hence, the introduction of *N*-heterocyclic carbene (NHC) ligands^13,25−30^ has eventually led to ferrous and ferric complexes that show excited-state electron-transfer reactivity of their triplet metal-to-ligand charge transfer (^3^MLCT) states^31−40^ and doublet ligand-to-metal charge transfer (^2^LMCT) states,^28,41−47^ respectively. In particular, the use of two tridentate scorpionate ligands, giving a facial attachment of a pair of three NHC ligands to iron, has been shown to be advantageous. The arrangement of strongly σ-donating NHC ligands resulted in an increased iron LMCT lifetime, from 500 fs of the archetypical Fe(III) complex K_3_[Fe(CN)_6_]^48^ to 2 ns for the Fe(III)NHC complex [Fe(phtmeimb)_2_](PF_6_) (phtmeimb = [phenyltris(3-methylimidazol-2-ylidene)borate]^−^).^28^ [Fe(phtmeimb)_2_](PF_6_) was also the first Fe complex resulting in the visible emission of an appreciable quantum yield (0.02) and demonstrated CT photoredox properties.^28^ Analogues of [Fe(phtmeimb)_2_](PF_6_), substituted with electron-donating and electron-withdrawing (EW) substituents in the para position of the phenyl-substituents of the scorpionate ligand did not show any impact on the properties of the analogues compare to the parent compound.^49^ For the iron MLCT state, the lifetime increased from <100 fs for the archetypical Fe(II) complex [Fe(bpy)_3_](PF_6_)_2_ (bpy = 2,2′-bipyridyl)^50^ to 0.5 ns for the Fe(II)NHC complex [Fe(btz)_3_](PF_6_)_2_ (btz = 3,3′-dimethyl-1,1′-bis(*p*-tolyl)-4,4′bis(1,2,3-triazol-5-ylidene)).^27^

Despite the outstanding photophysical properties of reported FeNHC complexes, the Fe(III) complexes are primarily useful as hole donors, and therefore they have a reversed electron flow compared to the traditional ruthenium(II) and iridium(III) complexes used as photosensitizers and photocatalysts. The Fe(III) oxidation state is due to the presence of six NHC moieties around the iron center, resulting in facile in situ oxidation of the original Fe(II) complex to the Fe(III) complex. Recently, however, there is one example of an Fe(II) phenylphenanthroline complex published that shows photoluminescence in the infrared region at room temperature, making promises for finally obtaining iron-based phosphorescence in the visible region.^51^ Hence, continuing to design and explore Fe(II)NHC complexes with the aim of matching the advantageous photophysical properties of Ru(II) and Ir(III) is thus a prioritized research area.

**Figure 1:**
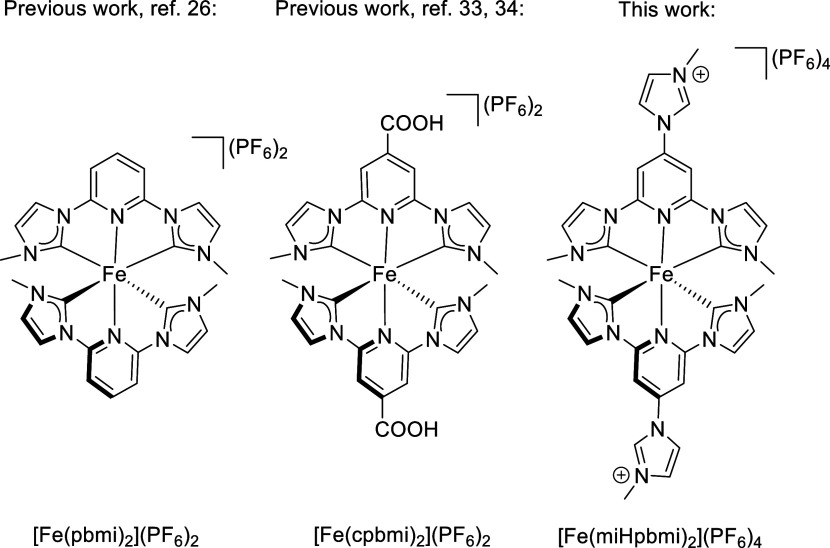
Chemical structure of [Fe(miHpbmi)_2_](PF_6_)_4_ investigated in this study compared with the parent complex [Fe(pbmi)_2_](PF_6_)_2_ and the related complex [Fe(cpbmi)_2_](PF_6_)_2_.

Following this line of research, we decided to investigate how substituents on the parent Fe(II)NHC complex [Fe(pbmi)_2_](PF_6_)_2_ (pbmi = 1,1′-(pyridine-2,6-diyl)bis(methylimidazol-2-ylidene)) (Figure 1),^25^ where all donor atoms of the two ligands are in a meridional arrangement, affect the photophysical and electrochemical properties of the complex. This ligand scaffold offers a robust complex that with its four NHC- and two pyridyl-moieties will stay in oxidation state +II and thus not be easily oxidized to Fe(III), as observed, for instance, when six NHC-moieties are included in the ligand system as for [Fe(phtmeimb)_2_](PF_6_) (see above).^28−30^ With carboxylic acid moieties attached to the ligand framework, the complex [Fe(cpbmi)_2_](PF_6_)_2_ (cpbmi = 1,1′-(4-carboxypyridine-2,6-diyl)bis(3-methylimidazol-2-ylidene)) has been formed (Figure 1) and resulted in a ^3^MLCT lifetime of 16–18 ps.^31,32^ This complex, despite its rather short excited-state lifetime, has been shown to partake in interfacial electron transfer and is thus the structural basis for utilizing FeNHC/s as photosensitizers in iron-based DSSCs.^6,34^

The increased MLCT lifetime of [Fe(cpbmi)_2_](PF_6_)_2_ compared to [Fe(pbmi)_2_](PF_6_)_2_ is a result of the introduction of the π-EW properties of the carboxylic acidic group. The effect of the introduction of a π-EW group has been investigated by Gros and co-workers, where the introduction of a protonated pyridyl group at the para-position of the pbmi ligand of the [Fe(pbmi)_2_](PF_6_)_2_ complex resulted in stabilization of the electron density over the π* orbitals in the MLCT state.^52^ Hence, the introduction of π-EW functionalities in the para-position of [Fe(pbmi)_2_](PF_6_)_2_ seems to be a promising way forward to increase the excited-state lifetime. However, care must be exercised due to the simultaneous reduced driving force for electron transfer to an external acceptor. An alternative π-EW ligand is the 3-methyl-1*H*-imidazole-3-ium-1-yl group, which in addition carries a full positive charge to further strengthen the EW properties. Furthermore, the 3-methyl-1*H*-imidazole-3-ium-1-yl group has no acidic hydrogen atoms, and thus, its properties are insensitive to pH, something that might be an advantage for future applications. Hence, the resulting complex [Fe(miHpbmi)_2_](PF_6_)_4_, where miHpbmi = 4-(3-methyl-1*H*-imidazol-3-ium-1-yl)(pyridine-2,6-diyl)bis(3-methylimidazol-2-ylidene) (Figure 1), should be a twin complex to [Fe(cpbmi)_2_](PF_6_)_2_ with pH-insensitive properties.

Here, we want to investigate how the π-EW 3-methyl-1*H*-imidazole-3-ium-1-yl group is influencing the photophysical and electrochemical properties of the parent complex [Fe(pbmi)_2_](PF_6_)_2_ when attached to the para-position of the pbmi ligand.

Following this line of research, we now report on the synthesis of the homoleptic Fe(II)NHC complex [Fe(miHpbmi)_2_](PF_6_)_4_ (Figure 1) having imidazolium groups on the para-position of the central pyridine rings. Moreover, the ground state was characterized by NMR, HR-MS, elemental analysis, scXRD analysis, and electrochemistry and quantum chemical calculations. The excited state was investigated by steady-state absorption spectroscopy, transient absorption spectroscopy, and quantum chemical calculations. The properties of the new complex were compared with the previously reported unsubstituted parent complex [Fe(pbmi)_2_](PF_6_)_2_ and the related complex Fe(cpbmi)_2_](PF_6_)_2_ (Figure 1).

## Result and Discussion

### Synthesis and Spectroscopic Characterizations

**Scheme 1:**
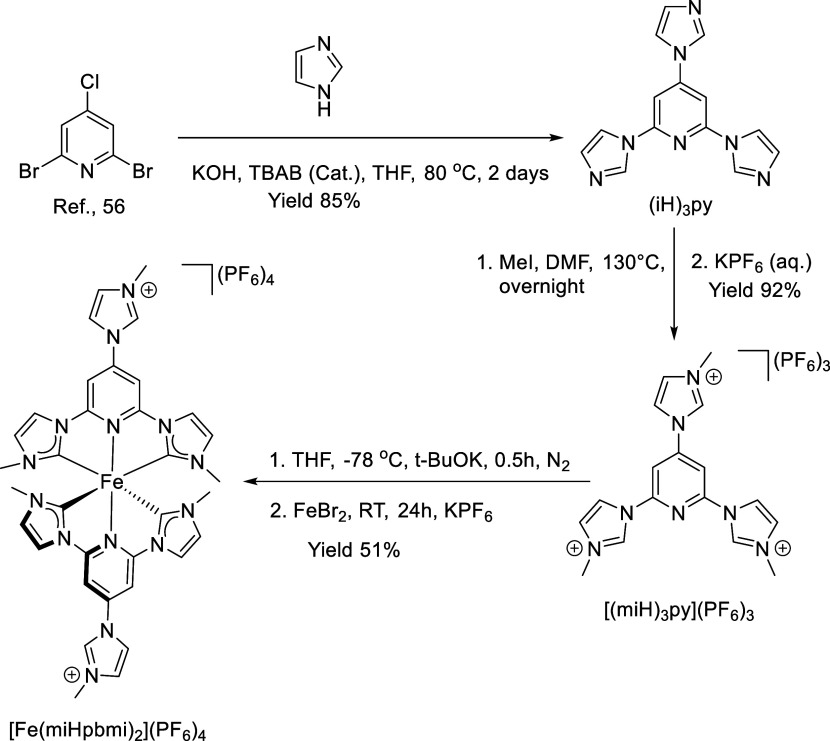
Synthesis of the [Fe(miHpbmi)_2_](PF_6_)_4_ Complex, Pre-NHC Ligand [(miH)_3_py](PF_6_)_3_, and Ligand (iH)_3_py

The C^∧^N^∧^C pincer pre-NHC ligand [(miH)_3_py](PF_6_)_3_ ((miH)_3_py = 2,4,6-tris(3-methyl-1*H*-imidazole-3-ium-1-yl)pyridine) was employed to synthesize [Fe(miHpbmi)_2_](PF_6_)_4_ with one additional imidazolium group at the 4-position of the central pyridine ring compared with the pre-NHC ligand [(miH)_2_py](PF_6_)_2_ ((miH)_2_py = 2,6-bis(3-methyl-1*H*-imidazole-3-ium-1-yl)pyridine) for the synthesis of the parent complex [Fe(pbmi)_2_](PF_6_)_2_. The synthesis of [Fe(miHpbmi)_2_](PF_6_)_4_ is shown in Scheme 1. The synthesis of [(miH)_3_py](PF_6_)_3_ is based on a slightly modified patented procedure^53^ and starts with 2,6-dibromo-4-chloropyridine that was reacted with imidazole and potassium hydroxide in the presence of a catalytic amount of tetrabutylammonium bromide (TBAB) to yield the trisubstituted ligand intermediate, 2,4,6-tris(imidazole-1*H*-yl)pyridine ((iH)_3_py). The C^∧^N^∧^C pincer pre-NHC ligand [(miH)_3_py](PF_6_)_3_ was then prepared through methylation of the (iH)_3_py synthetic intermediate. Due to the poorer reactivity of Fe^2+^ compared to Ru^2+^, Fe^2+^ must be reacted with the free carbene. Hence, the homoleptic Fe(II) complex [Fe(miHpbmi)_2_](PF_6_)_4_ was finally synthesized from FeBr_2_ from the free carbene NHC ligand (miH)_3_py, the latter generated in situ from [(miH)_3_py](PF_6_)_3_ using a strong sterically hindered base under the nitrogen atmosphere (for the protocol, see Supporting Information).

**Figure 2:**
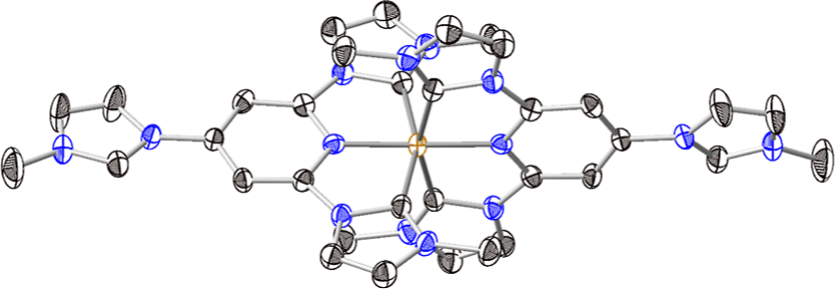
Molecular structure of the [Fe(miHpbmi)_2_](PF_6_)_4_ complex as determined by scXRD. Thermal ellipsoids are shown at a 30% probability. Hydrogen atoms, counterions, and solvent molecules are omitted for clarity. The displayed atoms are iron-orange; carbon-black, and nitrogen-blue.

The ^1^H NMR and ^13^C NMR spectra of the C^∧^N^∧^C pincer pre-NHC ligand [(miH)_3_py](PF_6_)_3_ and metal complex [Fe(miHpbmi)_2_](PF_6_)_4_ corresponded well with their proposed chemical structures. [Fe(miHpbmi)_2_](PF_6_)_4_ showed well-resolved ^1^H and ^13^C NMR signals, strongly supporting a low-spin iron d^6^ complex (for details, see Supporting Information). Wine-red single crystals of [Fe(miHpbmi)_2_](PF_6_)_4_ suitable for scXRD analysis were grown from a saturated acetonitrile solution of the complex by the slow diffusion of diethyl ether at room temperature. The molecular structure of [Fe(miHpbmi)_2_](PF_6_)_4_ was determined by scXRD and confirmed the Fe(II) oxidation state (Figure 2). The complex [Fe(miHpbmi)_2_](PF_6_)_4_ showed a distorted octahedral coordination geometry. The observed Fe–C_NHC_ (1.958(5)–1.961(5) Å) and Fe–N (1.906(4) Å) bond lengths and the bite angles C_NHC_–Fe–C_NHC_ (158.29(19)°) and N–Fe–N (178.9(2)°) slightly deviated from the reported unfunctionalized congener, [Fe(pbmi)_2_](PF_6_)_2_, bond lengths Fe–C_NHC_: 1.965(3)–1.970(3) Å; Fe–N: 1.919(3)–1.930(3) Å, bond angles C_NHC_–Fe–C_NHC_ 158.3(2)° and N–Fe–N 178.6(1)°. However, compared to the carboxylic acids congener [Fe(cpbmi)_2_](PF_6_)_2_, bond lengths Fe–C_NHC_: 1.943(10) Å; Fe–N: 1.905(7) Å are relatively shorter while C_NHC_–Fe–C_NHC_ bond angle 158.6(5)° resembles those of the [Fe(miHpbmi)_2_](PF_6_)_4_ and [Fe(cpbmi)_2_](PF_6_)_2_ complexes. Interestingly, the bond angle N–Fe–N was estimated to be 180.0(0)°, slightly expanded in comparison to those in the [Fe(miHpbmi)_2_](PF_6_)_4_ and [Fe(cpbmi)_2_](PF_6_)_2_ complexes (for details, see Supporting Information).^34^ It is noteworthy that both (iH)_3_py and [(miH)_3_py](PF_6_)_3_ were able to crystallize, and the resulting data was analyzed (for details, see Supporting Information).

### Mößbauer Spectrometry

**Figure 3:**
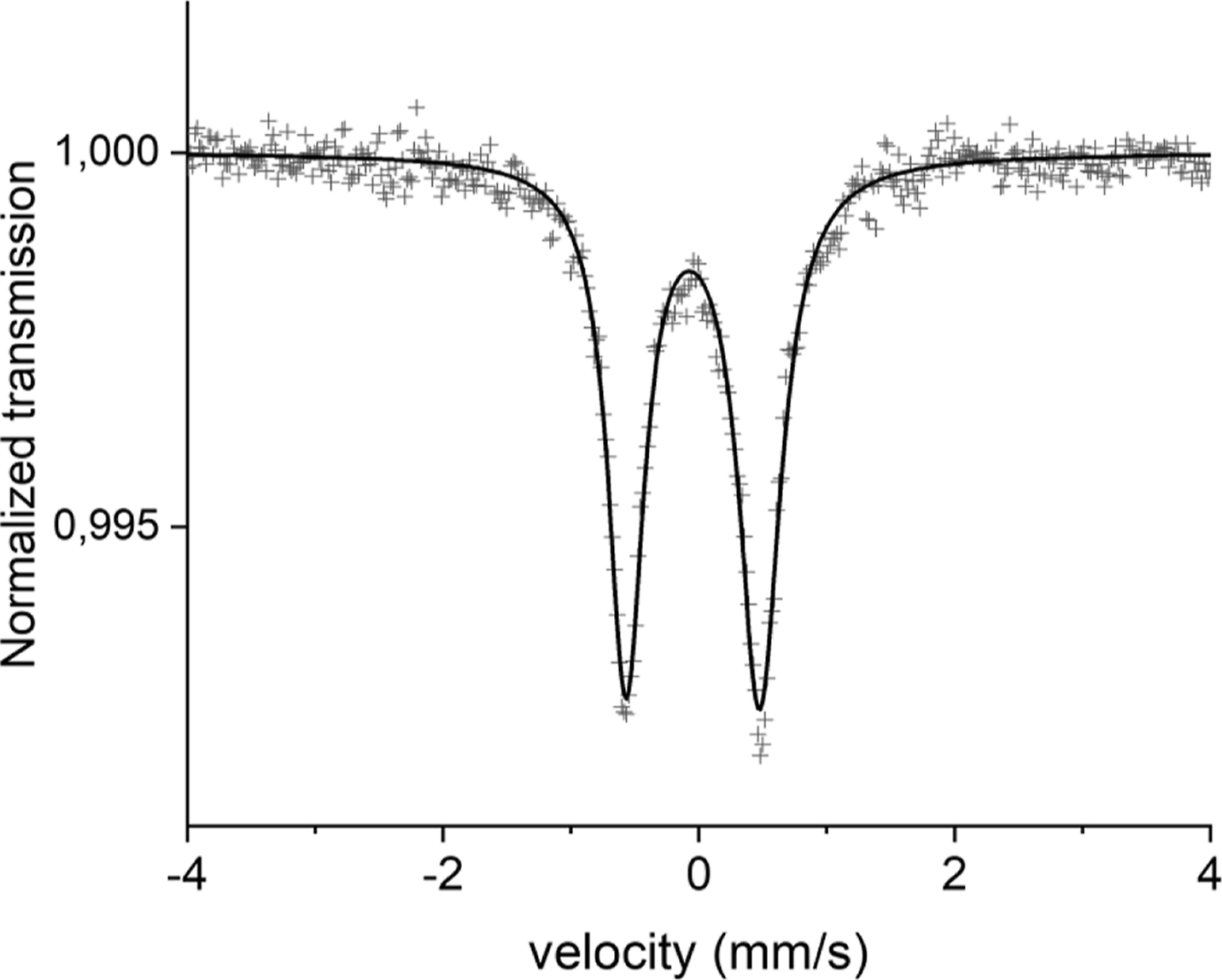
Mößbauer spectra of [Fe(miHpbmi)_2_](PF_6_)_4_ were recorded at 295 K.

Mößbauer spectra of [Fe(miHpbmi)_2_](PF_6_)_4_ at 295 and 85 K reveal a quadrupole split doublet structure with an area line intensity of A_+_/A_–_ = 1/0.83(4). The normal to the absorber plane had an angle α = 0° to the incoming γ-ray. The fitting results are presented in Figure 3 and Table 1. The results from the 295 and 85 K measurements are presented in Table 1.

**Table 1:** Results of the Fitting Procedure of the Mößbauer Spectra of [Fe(miHpbmi)_2_](PF_6_)_4_^a^

temperature (K)	CS (mm/s)	|QS| (mm/s)	Γ (mm/s)	A_–_/A_+_
295	–0.046(4)	1.05(1)	0.39(2)	0.83(4)
85	0.017(2)	1.13(1)	0.42(2)	0.93(2)

a CS is the center shift relative natural Fe held at 295 K, |QS| is the magnitude of the electric quadrupole splitting, Γ is the FWHM Lorentzian line width, and A_+_ and A_–_ are the line areas for the highest and lowest velocities of the doublet lines, respectively.

Comparison of the found center shifts CS and magnitude of electric quadrupole splittings |QS| for [Fe(miHpbmi)_2_](PF_6_)_4_ at 85 K is done with corresponding values found for other complexes measured in this type of Fe-complexes.^26,28,49,54^ With the present values, it seems quite clear that Fe in [Fe(miHpbmi)_2_](PF_6_)_4_ is of a low spin Fe(II) type. The found doublet line area asymmetry can be due to texture effects and/or anisotropic Debye–Waller factors.

### Electro- and Spectroelectrochemistry

**Figure 4:**
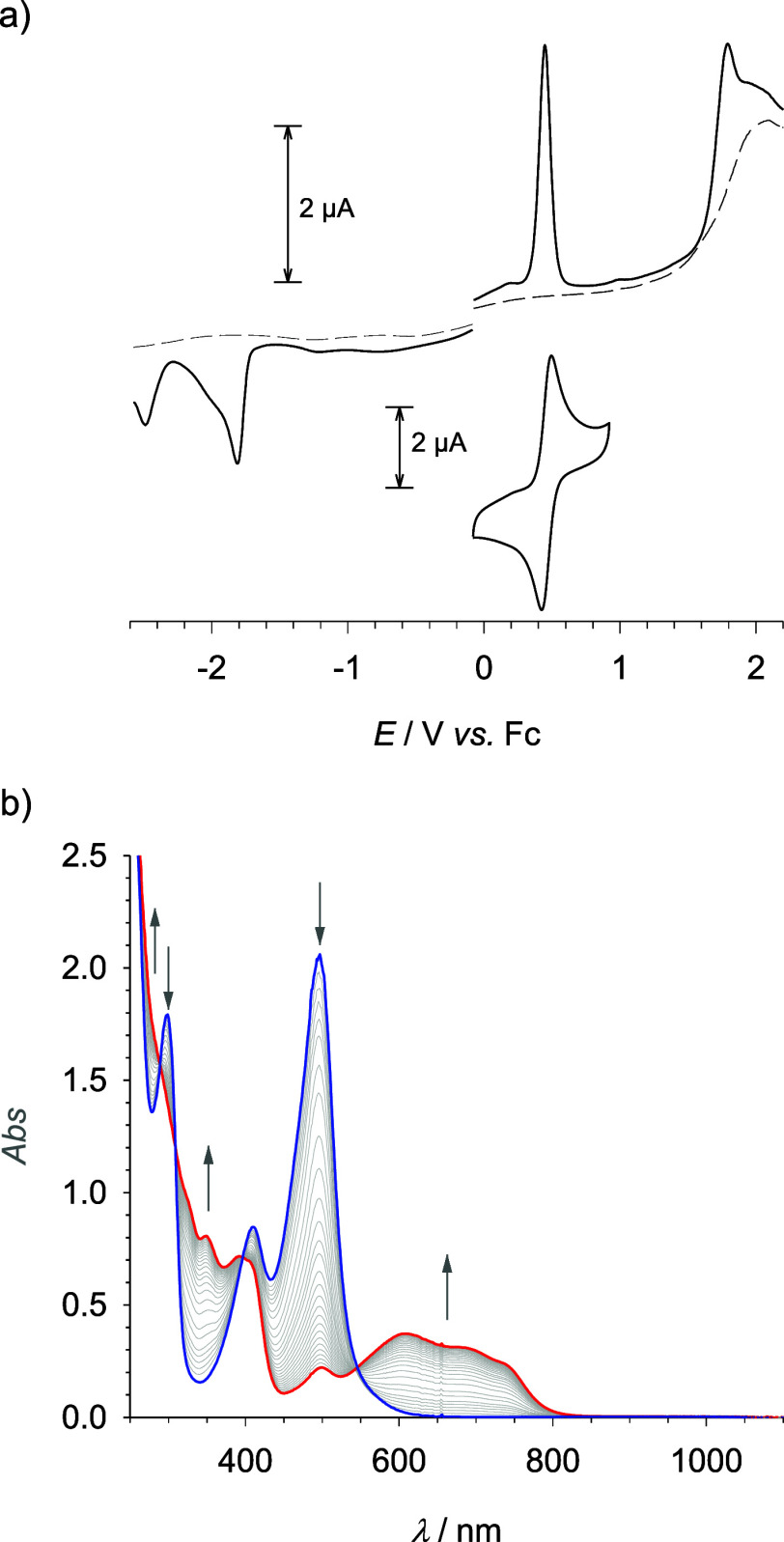
Electro- and spectroelectrochemistry of [Fe(miHpbmi)_2_](PF_6_)_4_ (1 mM) in acetonitrile with 0.1 M TBAPF_6_. (a) Differential pulse and cyclic voltammograms (50 mV s^–1^). (b) Electronic absorption spectrum (*I* = 1 mm) of [Fe(miHpbmi)_2_]^4+^ (blue —) and spectral changes upon oxidation (0.82 V) to [Fe(miHpbmi)_2_]^5+^ (red —).

Electrochemically, [Fe(miHpbmi)_2_](PF_6_)_4_ undergoes reversible one-electron oxidation with a half-wave potential of *E*_1/2_ = 0.46 V vs Fc (Figure 4a). In analogy to the parent complex and in agreement with the spectroelectrochemistry data (Figure 4b), the reversible oxidation can be attributed to the Fe(III/II) couple. Compared to the parent complex, [Fe(pbmi)_2_](PF_6_)_2_, the Fe(III/II) couple of [Fe(miHpbmi)_2_](PF_6_)_4_ shows the expected anodic shift caused by the electron-withdrawing effect of the methylimidazolium substituents (Table 2). The same effect causes a much more pronounced anodic shift of the first reduction to −1.81 V (Figures 4a and S13) in agreement with its assignment to a ligand-based process. The shifts of both potentials are similar to those induced by the carboxylic acid substituents in [Fe(cpbmi)_2_](PF_6_)_2_. This result demonstrates that the EW effects of methylimidazolium and carboxylic acid substituents are rather similar, despite the positive charge that the former introduces to the second coordination sphere. Comparing [Fe(cpbmi)_2_](PF_6_)_2_ to the parent unsubstituted complex, the diminished potential gap between metal oxidation and ligand reduction is in good agreement with the bathochromic shift of the lowest energy absorption band assigned to the MLCT transition. The trend predicted by the electrochemical data is also reflected by the even lower energy absorption of [Fe(cpbmi)_2_](PF_6_)_2_ (Table 2). The MLCT assignment is further supported by the bleaching of the 497 nm band upon oxidation of the metal center and the concomitant rise of low energy absorption extending to about 800 nm. The latter is in line with the expected lowest energy LMCT transition of the resulting Fe(III) complex based on the potentials of the Fe(III/II) couple and the presumably ligand-based oxidation at 1.79 V.

**Table 2:** Ground-State Absorption and Redox Properties for [Fe(miHpbmi)_2_](PF_6_)_4_ Compared to the Parent Complex [Fe(pbmi)_2_](PF_6_)_2_ and the Related Complex [Fe(cpbmi)_2_](PF_6_)_2_^g^

complex^a^	λ (nm)	*E* (eV)	ε·10^3^ (M^–^^1^ cm^–^^1^)	*E*_1/2_ Fe^III/II^ (V)^b^	*E*_p_ L^0/–^ (V)^b^^,^^c^
[Fe(pbmi)_2_](PF_6_)_2_^d^	457	2.71	15	0.31	–2.39
[Fe(miHpbmi)_2_](PF_6_)_4_	496	2.50	21	0.46	–1.81
[Fe(cpbmi)_2_](PF_6_)_2_^e^	520	2.38	25^f^	0.45	–1.71

a As PF_6_^–^ salt in acetonitrile (unless otherwise is noted).

b Vs Fc^+^/Fc in acetonitrile with 0.1 M TBAPF_6_.

c DPV peak potential of irreversible wave.

d From ref (25).

e From ref (32).

f From ref (36), measured in an acidic buffer (0.1 M TBA methanesulfonate and 0.1 M methanesulfonic acid in acetonitrile) to ensure that the complex is protonated.

g Extinction coefficients (ε) at peak wavelength (λ) and peak energy (*E*), potentials of first oxidation (*E*_1/2_ Fe^III/II^) and first reduction (*E*_p_ L^0/–^).

### Steady-State Absorption

**Figure 5:**
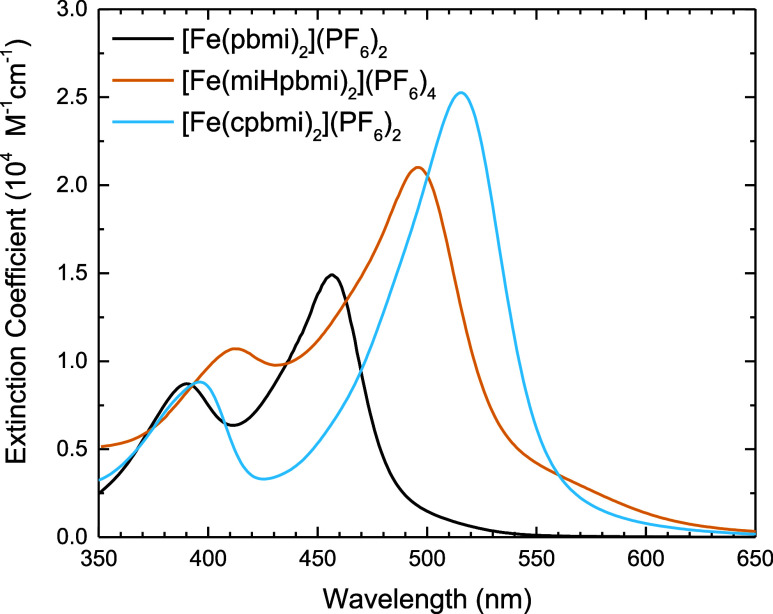
Extinction coefficient for the complex [Fe(miHpbmi)_2_](PF_6_)_4_, plotted together with the extinction coefficient of the parent complex [Fe(pbmi)_2_](PF_6_)_2_ and the related complex [Fe(cpbmi)_2_](PF_6_)_2_.

The steady-state absorption of [Fe(miHpbmi)_2_](PF_6_)_4_ in acetonitrile is compared to those of the parent complex [Fe(pbmi)_2_](PF_6_)_2_ and the related complex [Fe(cpbmi)_2_](PF_6_)_2_ in Figure 5. The wavelength region shown in Figure 5 is attributed to the MLCT absorption band, based on previous assignments^25^ and time-dependent density functional theory (TD-DFT) calculations (Table S8). The MLCT absorption band of [Fe(miHpbmi)_2_](PF_6_)_4_ has a double-peak structure similar to the parent complex. However, for [Fe(miHpbmi)_2_](PF_6_)_4_ both peaks are red-shifted, and also a shoulder at ∼460 nm is more pronounced. The red shift is attributed to the π-EW properties of the miHpbmi-ligand, having a stabilizing effect on the ligand-centered π*-orbitals, something however more pronounced in [Fe(cpbmi)_2_](PF_6_)_2_ that has the highest red shift, demonstrating that the CO_2_H group is more π-EW than 3-methyl-1*H*-imidazole-3-ium-1-yl. The positively charged miHpbmi-ligand is, however, stabilizing both HOMO and LUMO (see Figure S25). Therefore, the absorption of [Fe(miHpbmi)_2_](PF_6_)_4_ is slightly less red-shifted compared to [Fe(cpbmi)_2_](PF_6_)_2_. Overall, the extinction of [Fe(miHpbmi)_2_](PF_6_)_4_ is higher than that of the parent complex [Fe(pbmi)_2_](PF_6_)_2_. This is most probably due to the extension of the ligand π*-system from the imidazolium substituent; however, the extinction is not as high as for [Fe(cpbmi)_2_](PF_6_)_2_, which has the highest extinction, demonstrating at least a significantly larger extension of the π-system by CO_2_H, compared to 3-methyl-1*H*-imidazole-3-ium-1-yl. The steady-state photophysical properties are concluded in Table 2.

### Transient Absorption Spectroscopy

**Figure 6:**
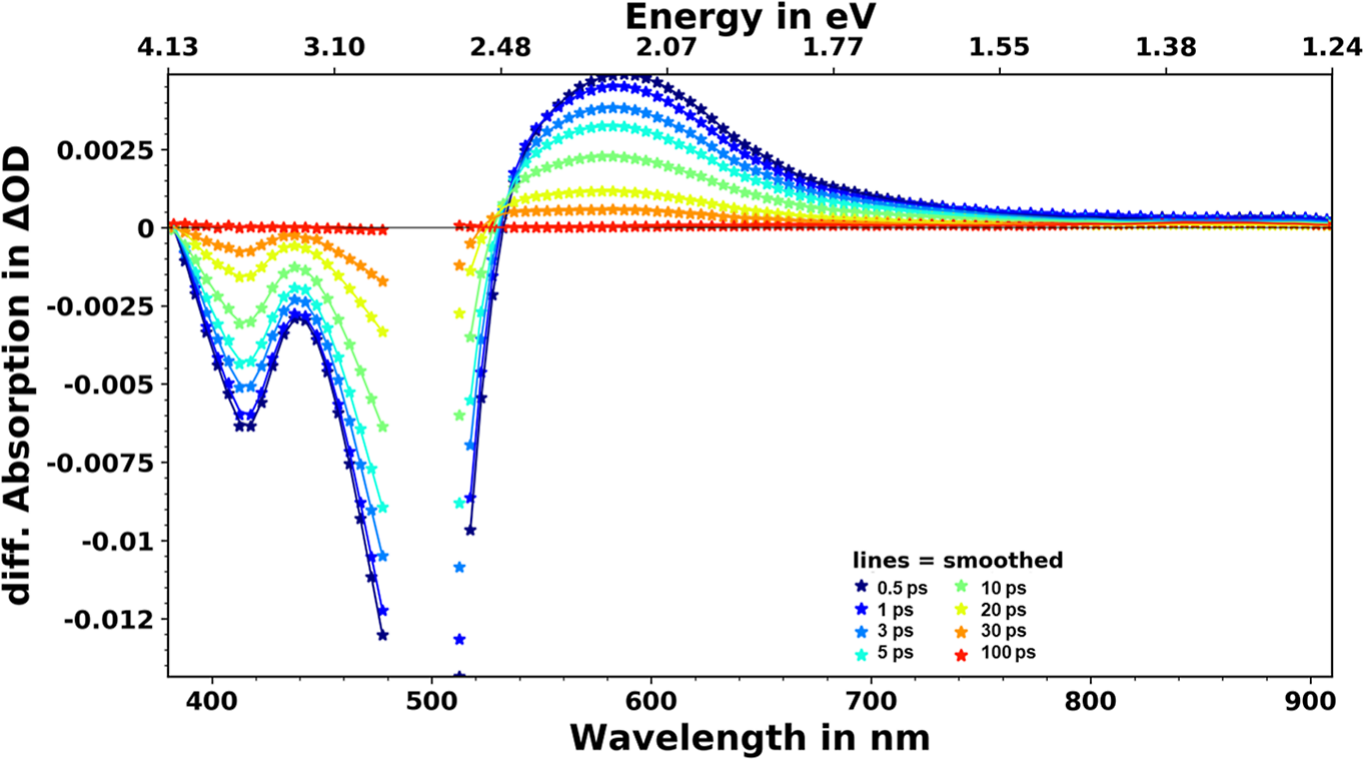
Transient absorption spectra of [Fe(miHpbmi)_2_](PF_6_)_4_ in acetonitrile after excitation at 500 nm at selected delay times. Spectra have been chirp- and background-corrected and also cut to remove excitation scatter.

Transient absorption spectroscopy was measured for [Fe(miHpbmi)_2_](PF_6_)_4_ in acetonitrile, and the spectral evolution after excitation at 500 nm is seen in Figure 6. In the ∼380–520 nm region, there is a negative band well corresponding to the inverted steady-state absorption (Figure 6), attributed to ground-state bleach (GSB). On the red side of this band is the excited-state absorption (ESA), which peaks at ∼580 nm. The ESA band extends further into the blue for longer delay times, also seen as a tiny blue shift of the ESA peak. We relate this spectral shift to intramolecular vibrational relaxation and solvation. Solvent cooling in the excited state often takes place on the ps-time scale and results in a narrowing of the ESA bandwidth, which could also influence the observed ESA spectral evolution but should not have the dominant contribution. This assignment is supported by observations of very similar ESA dynamics in other solvents where solvent viscosity and polarity were substantially varied (see Figures S18–S20). Furthermore, the overall decay of the ESA is nearly independent of the solvents used (see Figure S21). The decay of ESA in DMSO appears slightly slower than in all other solvents, which could indicate that solvent viscosity may influence the depopulation of the excited state.

**Figure 7:**
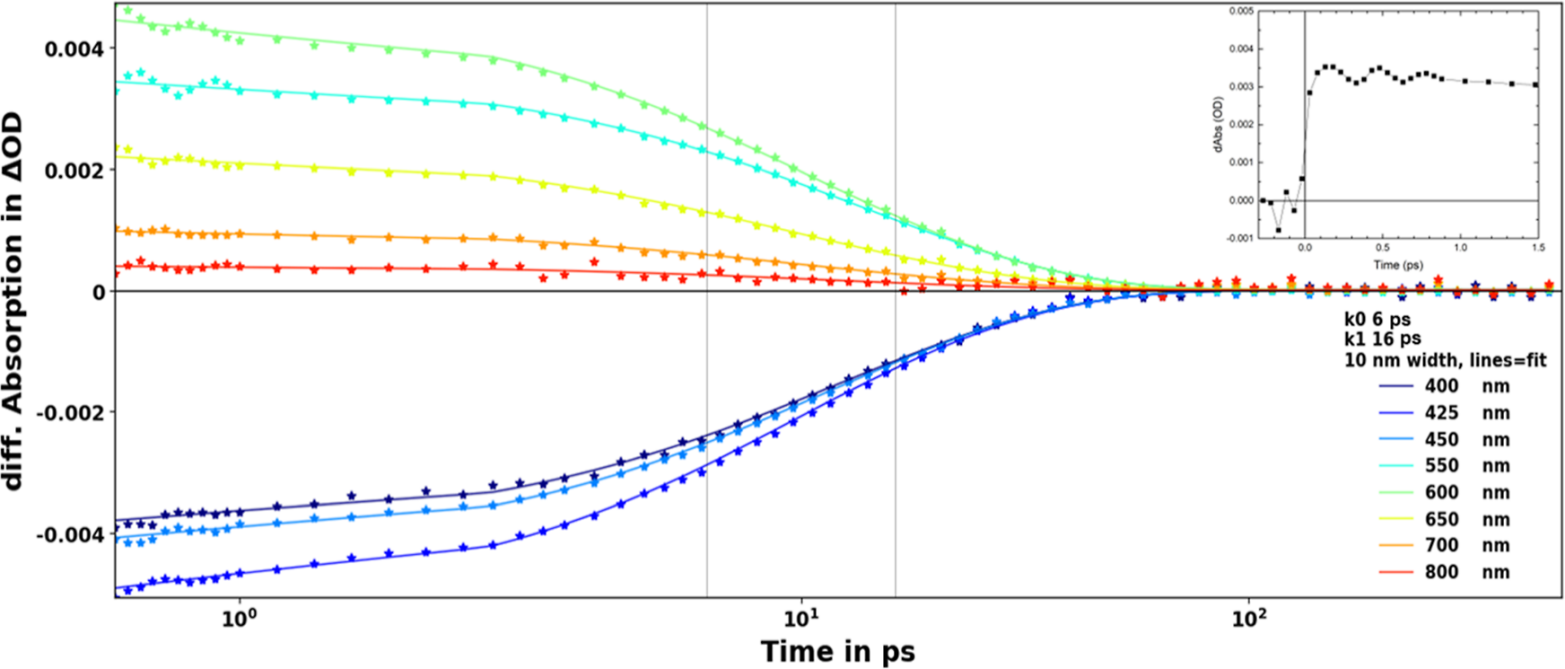
Selected transient absorption kinetics of [Fe(miHpbmi)_2_](PF_6_)_4_ in acetonitrile after excitation at 500 nm. Lines represent the fit to the data resulting from global analysis with two decay components (6 and 16 ps). Kinetics have been chirp- and background-corrected. In the inset, a kinetic at 550 nm is plotted up to 1.5 ps in order to see the strong oscillations.

[Fe(miHpbmi)_2_](PF_6_)_4_ in acetonitrile shows two components after global fitting of the transient absorption dynamics starting from a delay time of 0.5 ps. The major lifetime component is 16 ps, and the minor component is 6 ps. Selected kinetics with a global fit are shown in Figure 7. The decay-associated spectra (Figure S16) of the two components are very similar, and both resemble the fully developed transient absorption spectra with GSB and ESA (Figure 6). The longer component (16 ps) is a bit blue-shifted compared to the shorter one (6 ps), in line with observation of the spectral evolution. Since both components have a very similar spectrum, it is likely that they can be associated with the same state. Both also have the characteristic GSB band, which implies that they both reflect repopulation of the ground state. The thermalized excited state then decays to the ground state with a lifetime of 16 ps. Global fit of the data measured in other solvents is presented in Figure S22. Minor differences in the obtained decay components are most probably indicative of experimental errors reflected in the uncertainty in the fits. The main conclusion from the time-resolved measurements of [Fe(miHpbmi)_2_](PF_6_)_4_ is the clear similarity in overall dynamics and close agreement for the excited-state lifetime with the [Fe(pbmi)_2_](PF_6_)_2_ parent complex.^25^

In Figure 7 at very early delay times, oscillations are present at virtually all probed wavelengths. The more pronounced oscillations at 550 nm are shown in the inset of Figure 7. Nearly identical oscillations were observed in all solvents used (Figure S23). The oscillations at 550 nm in acetonitrile were fitted by a damped cosine function with a period time of 310 fs and a damping time of 1.1 ps; see Figure S17. In other solvents, the fitting of oscillations resulted in very similar fit parameters, supporting the assignment of the oscillations to the intramolecular type of coherent motion. Similar oscillations have been seen before in transient absorption kinetics for the parent complex [Fe(pbmi)_2_](PF_6_)_2_.^25^ Furthermore, in a time-resolved X-ray study of [Fe(pbmi)_2_](PF_6_)_2_, the authors identified oscillations with a 278 fs period time and 500 fs damping time that were ascribed to Fe-ligand bond length stretching through a partial population of the ^3^MC state.^55^ In [Fe(miHpbmi)_2_](PF_6_)_4_, the oscillation period is a bit longer and the damping time is substantially longer. Although the similarity of the oscillatory behavior in the two complexes is striking, the nature of the oscillations in [Fe(miHpbmi)_2_](PF_6_)_4_ cannot be determined with the transient absorption technique alone. We tentatively associate the oscillations with at least a partial population of the ^3^MC state.

### Quantum Chemical Calculations

**Figure 8:**
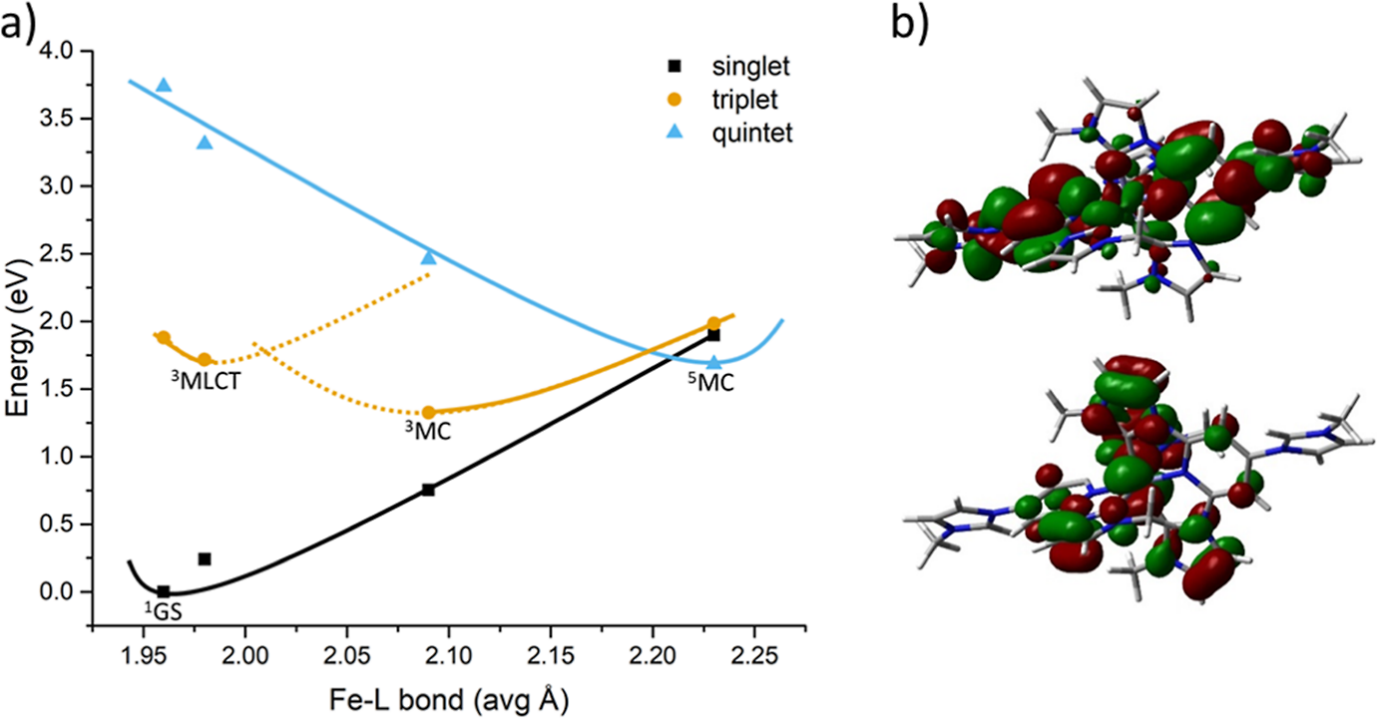
(a) Projected potential energy surfaces for [Fe(miHpbmi)_2_](PF_6_)_4_. Key optimized states are named, and other points were obtained from single point calculations at the selected geometry. Solid and dotted lines represent the authors’ interpretation. (b) Contour plots of Kohn–Sham HOMO (bottom) and LUMO (top) for the optimized ^1^GS.

Key electronic states of [Fe(miHpbmi)_2_](PF_6_)_4_, including the singlet ground state (^1^GS), triplet metal-to-ligand charge transfer (^3^MLCT) state, triplet metal-centered (^3^MC) state, and quintet metal-centered (^5^MC) state were first obtained through full, unconstrained, geometry optimizations using restricted or unrestricted DFT optimizations as appropriate. The level of theory used was B3LYP* with the basis set 611G(d) modeled in an empirical solvent model of acetonitrile. The electronic structure character of each state was confirmed by the spin density distribution of each of the open-shell states (see Figure S24 and Table S6). The ^3^MLCT state is similar in geometry to the ^1^GS, whereas MC states are associated with a large Fe-ligand bond elongation. The projected potential energy surfaces of these states in the Fe-ligand bond coordinate are listed in Figure 8a. These calculations confirm the basic similarity of the electronic states in this system to previous reports for the parent complexes [Fe(pbmi)_2_](PF_6_)_2_ and [Fe(cpbmi)_2_](PF_6_)_2_,^56^ including the presence of a local minimum for the ^3^MLCT state, as well as ^3^MC states that are significantly destabilized compared to traditional iron polypyridyl complexes. The energy difference between the ^3^MLCT and ^3^MC minima is ∼0.4 eV for [Fe(miHpbmi)_2_](PF_6_)_4_ (Figure 8a).

The electronic structure, represented by contour plots of the highest occupied molecular orbital (HOMO) and lowest unoccupied molecular orbital (LUMO) for the relaxed ^1^GS in Figure 8b, shows a significant direct influence of the imidazolium side groups on the frontier molecular orbital structure, in particular, in terms of strong mixing with the LUMO π* level. The HOMO–LUMO transition reflects an MLCT transition where the electron density is relocated symmetrically to the outer part of the ligands. In Figure S25, we see that for [Fe(miHpbmi)_2_](PF_6_)_4_ both HOMO and LUMO are stabilized compared to those of the parent complex. The LUMO is however stabilized more, which leads to a net lowering of the HOMO–LUMO gap seen as a red shift in the absorption spectrum. The positive charge on the miHpbmi-ligand helps to stabilize the electron density both when residing on the metal (in GS and MC-states) and on the ligand (MLCT-states). For [Fe(cpbmi)_2_](PF_6_)_2_, mainly the LUMO is stabilized compared to [Fe(pbmi)_2_](PF_6_)_2_ (see Figure S25), which explains the larger red shift of the absorption for this molecule.

The nature of the optical absorption bands in the steady-state absorption spectrum was also considered based on results from TD-DFT calculations of singlet–singlet excitation from the optimized singlet ground state of [Fe(miHpbmi)_2_](PF_6_)_4_ (see Table S8). The TD-DFT calculations show that the main experimental bands in the visible part of the spectrum with wavelengths longer than ca. 350 nm corresponds to a set of calculated transitions with MLCT character mainly involving the HOMOs with significant t_2g_ character going to the LUMOs with mainly ligand π* character. Approaching 300 nm, ligand-centered transitions start to appear, and below 290 nm these are the dominating transitions.

## Conclusions

We report a new homoleptic Fe(II) C^∧^N^∧^C pincer NHC complex [Fe(miHpbmi)_2_](PF_6_)_4_. The synthetic strategy, taking advantage of the electron-withdrawing methylimidazole substituent in the ligand, enhanced the excited-state lifetime almost twice compared to that of the parent complex [Fe(pbmi)_2_](PF_6_)_2_. In comparison to the parent complex, the Fe(III/II) couple and the ligand reduction potential show anodic shifts due to the electron-withdrawing group. This leads to a red-shifted absorption spectrum compared to the parent complex, since the effect of stabilizing the ligand-centered LUMO is higher than that of stabilizing the MC HOMO. Furthermore, the extinction of [Fe(miHpbmi)_2_](PF_6_)_4_ is higher than the parent complex probably owing to the extension of the ligand π*-system by the imidazolium substituent. The imidazolium group was overall shown to yield photophysical properties similar to those of the carboxylic group when implemented as a substituent on the [Fe(pbmi)_2_](PF_6_)_2_ parent complex. [Fe(miHpbmi)_2_](PF_6_)_4_ thus serves as a pH-insensitive analogue to [Fe(cpbmi)_2_](PF_6_)_2_. To further improve the photophysical properties, such as the excited-state lifetime and broader absorption spectrum, our group aims to modify the [Fe(pbmi)_2_](PF_6_)_2_ ligand scaffold by introducing other substituents.

## Supporting Information Available

Synthesis, ^1^H and ^13^C NMR spectra, HR-MS spectra, single-crystal X-ray diffraction, Mößbauer spectroscopy, steady-state absorption spectroscopy, transient absorption spectroscopy, quantum chemistry, and x-ray crystallographic data for [Fe(miHpbmi)_2_](PF_6_)_4_ (PDF)
