# Correction to “Tailoring the Photophysical Properties of a Homoleptic Iron(II) Tetra *N*-Heterocyclic Carbene Complex by Attaching an Imidazolium Group to the (C^∧^N^∧^C) Pincer Ligand—A Comparative Study”

**DOI:** 10.1021/acs.inorgchem.4c01017

**Published:** 2024-04-02

**Authors:** Om Prakash, Linnea Lindh, Arvind Kumar Gupta, Yen Tran Hoang Hai, Nidhi Kaul, Pavel Chábera, Fredrik Lindgren, Tore Ericsson, Lennart Häggström, Daniel Strand, Arkady Yartsev, Reiner Lomoth, Petter Persson, Kenneth Wärnmark

**Affiliations:** † Centre for Analysis and Synthesis, Department of Chemistry, Lund University, Box 124, Lund SE-22100, Sweden; ‡ Chemical Physics Division, Department of Chemistry, Lund University, Box 124, Lund SE-22100, Sweden; § Theoretical Chemistry Division, Department of Chemistry, Lund University, Box 124, Lund SE-22100, Sweden; ∥ Department of Chemistry—Ångström Laboratory, Uppsala University, Box 523, Uppsala SE-751 20, Sweden; ⊥ Department of Physics—Ångström Laboratory, Uppsala University, Box 523, Uppsala SE-751 20, Sweden

We correct the author list by adding the missing shared authorship between Om Prakash, Linnea Lindh, and Arvind Kumar Gupta.

