# Supplementary material for: Tailoring the Photophysical Properties of a Homoleptic Iron(II) Tetra N-Heterocyclic Carbene Complex by Attaching an Imidazolium Group to the (C∧N∧C) Pincer Ligand—A Comparative Study
Source: Inorg Chem. 2024 Feb 1;63(6):2909–18. doi: 10.1021/acs.inorgchem.3c02890 (PMC10865346; doi:10.1021/acs.inorgchem.3c02890)
Supplement: Supplementary file 1 — ic3c02890_si_001.pdf [file ic3c02890_si_001.pdf]

SUPPORTING INFORMATION

Tailoring the Photophysical Properties of a Homoleptic Iron (II) tetra N-Heterocyclic carbene complex by Attaching an Imidazolium Group to the (C^N^C) Pincer ligand – A Comparative Study

Om Prakash,[+a] Linnea Lindh,[+b,c] Arvind Kumar Gupta,[+a] Yen Tran Hoang Hai [b], Nidhi Kaul,[d] Pavel Chábera,[b] Fredrik Lindgren,[d] Tore Ericsson,[e] Lennart Häggström,[e] Daniel Strand,[a] Arkady Yartsev,[b,] Reiner Lomoth,[d,]* Petter Persson,[c,]* Kenneth Wärnmark[a,]**

+ shared authorship

Corresponding Authors

*A.Y.: E-mail: arkady.yartsev@chemphys.lu.se

*R.L.: E-mail: reiner.lomoth@kemi.uu.se.

*P.P.: E-mail: petter.persson@teokem.lu.se.

*K.W.: E-mail: kenneth.warnmark@chem.lu.se.

List of contents **Page**

S1. Synthesis and structure identifications……………………………………………2-12

S2. Single Crystal X-ray diffraction……………………………………………………12-20

S3. Mößbauer spectroscopy……………………………………………………………...20

S4. Electrochemistry……………………………………………………………………. .21

S5. Steady State Absorption Spectroscopy……………………………………………..22-23

S6. Transient absorption spectroscopy…………………………………………...….23-29

S7. Quantum Chemistry……………………………………………………………...29-33

S8. References…………………………………………………………………………33-34

S1. Synthesis and structure identifications

All commercial reagents and solvents were used as received unless otherwise stated. ^{1}H and ^{13}C NMR spectra were recorded on a Bruker Avance II 400 MHz NMR spectrometer. Chemical shifts (δ) are reported to the shift-scale calibrated with the residual NMR solvent; CD_3CN (1.94 ppm for ^{1}H NMR spectra). Electrospray ionization–high resolution mass spectrometry (ESI–HRMS) and atmospheric pressure chemical ionization (APCI) for mass spectrometry were recorded on a Waters Micromass Q-Tof micro mass spectrometer. Infrared spectra were recorded as the neat compound on a Bruker Alpha-P FTIR spectrometer. Melting points of the compounds were measured on a Stuart Scientific Melting Point Apparatus-SMP3 and were corrected using standard substances. Elemental analyses were performed by Mikroanalytisches Laboratorium KOLBE (Mülheim an der Ruhr, Germany). Size of the bio-beads column is 120 cm in length and 4.5 cm in width.

Potassium *tert*-butoxide (1 M solution in THF) was purchased from Aldrich. Anhydrous ferrous chloride ($FeBr_2$) and potassium hexafluorophosphate (KPF_6) were purchased from Acros. 2,6-dibromo-4-chloropyridine was purschased from Sigma-Adrich. THF (Honeywell) was dried over Na/benzophenone and was subsequently distilled under argon prior to use. Anhydrous CH_3CN and diethyl ether was obtained from a PureSolv PSM-768 and Braun SPS-800 system respectively.

2,4,6-Tris(imidazol-1*H*-yl)pyridine ((iH)$_3$py). 2,6-Dibromo-4-chloropyridine (1.36 g, 5.00 mmol), imidazole (1.36 g, 20.0 mmol), KOH (1.72 g, 20.0 mmol) and tetrabutylammonium bromide (0.55 mmol) were mixed in 30 mL of THF. The reaction mixture was stirred at 80°C for 3 days. After cooling at the room temperature, the reaction mixture was diluted in water (100 mL) and extracted in DCM (3 x 50 ML) followed by washing saturated aqueous Na_2CO_3 solution (3 x 50 mL) and brine (100 mL). The combined organic phases were dried over

$MgSO_4$, filtrated, and concentrated under reduced pressure to get the product as a white solid (1.17 g, Yield: 85%); Mp: 256 °C. ^{1}H NMR (400 MHz, CD_3OD, 5 mM): δ (ppm) 8.77 (s, 2H, H_{Im}), 8.68 (s, 1H, H_{Im}), 8.12-7.11 (m, 2H, H_{Im}), 8.05 (s, 1H, H_{Im}), 8.00 (s, 2H, H_{Py}), 7.29-7.28 (m, 1H, H_{Im}), 7.22-7.21 (m, 2H, H_{Im}); ^{13}C{^{1}H} NMR (100.3 MHz, CD_3OD, 5 mM): δ (ppm) 149.7 (C_{Py}), 148.8 (C_{Py}), 136.1 (C_{Im}), 135.7 (C_{Im}), 130.1 (C_{Im}), 129.5 (C_{Im}), 117.6 (C_{Im}), 116.9 (C_{Im}), 100.1 (C_{Im}). APCI–HRMS (m/z): $[(C_{14}H_{11}N_7)+H]^+$ calcd for $C_{14}H_{11}N_7$, 278.1149; found, 278.1162; Elemental analysis: (%calculated, % found for $C_{14}H_{11}N_7 \cdot 0.7H_2O$): C (58.00, 58.32), H (4.31, 4.33), N (33.82,33.67).

2,4,6-tris(3-methyl-1*H*-imidazol-3-ium-1-yl)pyridine tris(hexafluorophosphate) ([(miH)$_3$py](PF$_6$)$_3$). To a solution of 2,4,6-tris(imidazol-1*H*-yl)pyridine (0.81 g, 2.0 mmol) in DMF (15 ml), iodomethane (2.84 g, 20.0 mmol) was added and stirred at 130 ºC overnight. After cooling at room temperature, a saturated solution of KPF_6 was added to precipitate the product. The resulting precipitate was washed with distilled water and dried under vacuum to afford the white solid as a product (1.39 g, Yield: 92%); Mp: 273 °C. ^{1}H NMR (400 MHz, CD_3CN, 7 mM): δ (ppm) 9.58 (s, 2H, H_{Im}), 9.29 (s, 1H, H_{Im}), 8.24-8.23 (m, 2H, H_{Im}), 8.12 (s, 2H, H_{Py}), 8.08-8.07 (m, 1H, H_{Im}), 7.23-7.22 (m, 1H, H_{Im}), 7.70-7.68 (m, 2H, H_{Im}), 4.05 (s, 9H, H_{Im}); ^{13}C{^{1}H} NMR (100.3 MHz, CD_3CN, 7 mM): δ (ppm) 147.5 (C_{Im}), 147.2 (C_{Im}), 136.1 (C_{Im}), 136.3 (C_{Py}), 135.8 (C_{Py}), 125.9 (C_{Im}), 125.7 (C_{Im}), 121.1 (C_{Im}), 119.5 (C_{Im}), 107.6 (C_{Im}), 55.2 (C_{Im}), 37.1 (C_{Im}). APCI–HRMS (m/z): $[(C_{17}H_{20}N_7)-(2PF_6)]^+$ calcd for $C_{17}H_{19}F_{12}N_7P_2$, 612.1058; found, 612.1079; Elemental analysis: (% calculated, % found for $C_{17}H_{20}F_{18}N_7P_3$): C (26.96, 26.59), H (2.66, 2.69), N (12.95, 12.78).

Iron bis[(4-(3-methyl-1H-imidazol-3-ium-1-yl)(pyridine-2,6-diyl)bis(3-methyl-imidazol-2-ylidene)] tetra(hexafluorophosphate) ([Fe(miHpbmi)$_2$](PF$_6$)$_4$). 2,4,6-Tris(3-methyl-1*H*-imidazol-3-ium-1-yl)pyridine tris(hexafluorophosphate) (0.757 g, 0.597 mmol) was vacuum-dried at 80 ºC overnight in a Schlenk tube. Dry THF (30 mL) was charged under N_2. The suspension was then cooled down to −78 ºC and *t*-BuOK in THF (3.5 mL, 3.5 mmol) was added dropwise and the reaction mixture was stirred for 30 min at −78 ºC. The cooling bath was removed and anhydrous $FeBr_2$ (0.118 g, 0.547 mmol) in 20 mL dry THF was injected to the Schlenk tube containing the in-situ generated carbene ligand solution with a syringe before the mixture warmed to room temperature under N_2. The resulted solution was stirred in dark under N_2 gas at room temperature for 24 hrs. The solution was filtered using pad of celite and the collected solid on celite pad was extracted with CH_3CN. The collected extraction was filtered and washed with CH_3CN. The resulting filtrate was evaporated to dryness and the resulting dark brown residue was dissolve in small amount of water and treated with aqueous solution of KPF_6 (0.195 g, 1.06 mmol). The wine-red precipitate was collected and washed with water. The resulting wine-red residue was dissolved in 10 mL of acetonitrile and the product was precipitated by addition of dry diethyl ether (100 mL). The wine-red precipitate was dissolved in a minimum amount of CH_3CN. The solution was filtered through a syringe filter w/ 0.2 μm PTFE membrane and the compound was purified on a Bio-Beads S-X1 size-exclusion chromatography column. The product was eluted using CH_3CN/toluene (1:1) as the eluent. The fraction containing the product was evaporated to dryness under vacuum. The wine-red colour single crystals suitable for X-ray diffraction were grown by slow diffusion of diethyl ether into the CH_3CN solution of the product (0.325 g, Yield: 51%), Mp: above 300 °C. ^{1}H NMR (400 MHz, CD_3CN, 20 mM): δ (ppm) 9.19 (s, 2H, H_{Im}), 8.01-8.00 (m, 10H, H_{Im}, H_{Py}), 7.75-7.74 (s, 2H, H_{Im}), 7.08-7.07 (m, 4H, H_{Im}), 4.08 (s, 6H, H_{Im}), 2.56 (s, 12H, H_{Im}); ^{13}C{^{1}H} NMR (100.3 MHz, CD_3CN, 20 mM): δ (ppm) 199.7 (C_{Im}), 154.5 (C_{Im}), 142.4 (C_{Im}), 136.1

(C_{Py}), 127.5 (C_{Py}), 125.5 (C_{Im}), 121.3 (C_{Im}), 117.1 (C_{Im}), 116.2 (C_{Im}), 98.7 (C_{Im}), 36.9 (C_{Im}), 34.2 (C_{Im}). APCI–HRMS (m/z): $[(C_{34}H_{36}F_{18}FeN_{14}P_3)-(PF_6)]^+$ calcd for $C_{34}H_{36}F_{18}FeN_{14}P_3$, 1131.1522; found, 1131.1525; Elemental analysis: (% calculated, % found for $C_{34}H_{36}F_{24}FeN_{14}P_4$): C (31.99, 31.91), H (2.84, 2.87), N (15.36, 15.34).

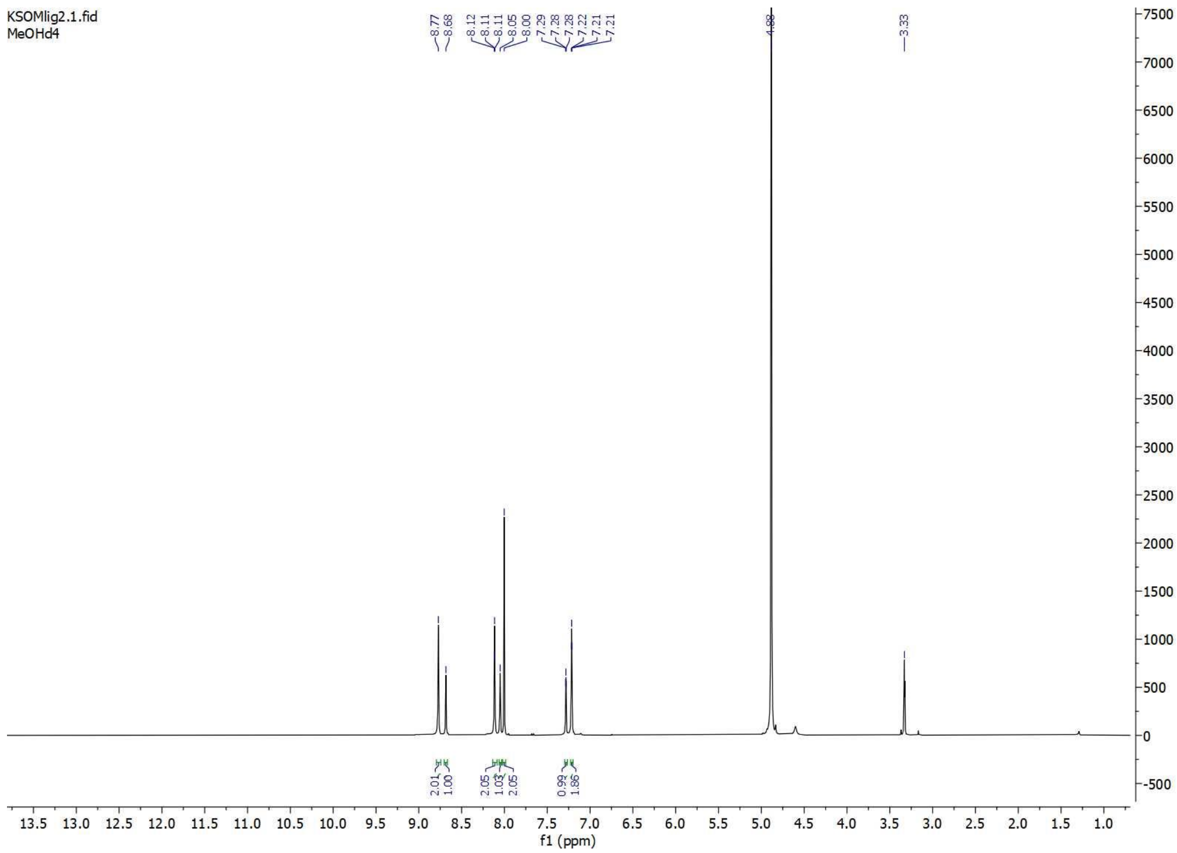

Figure S1. ^{1}H NMR spectrum of the $(iH)_3py$ ligand precursor in CD_3OD (5 mM).

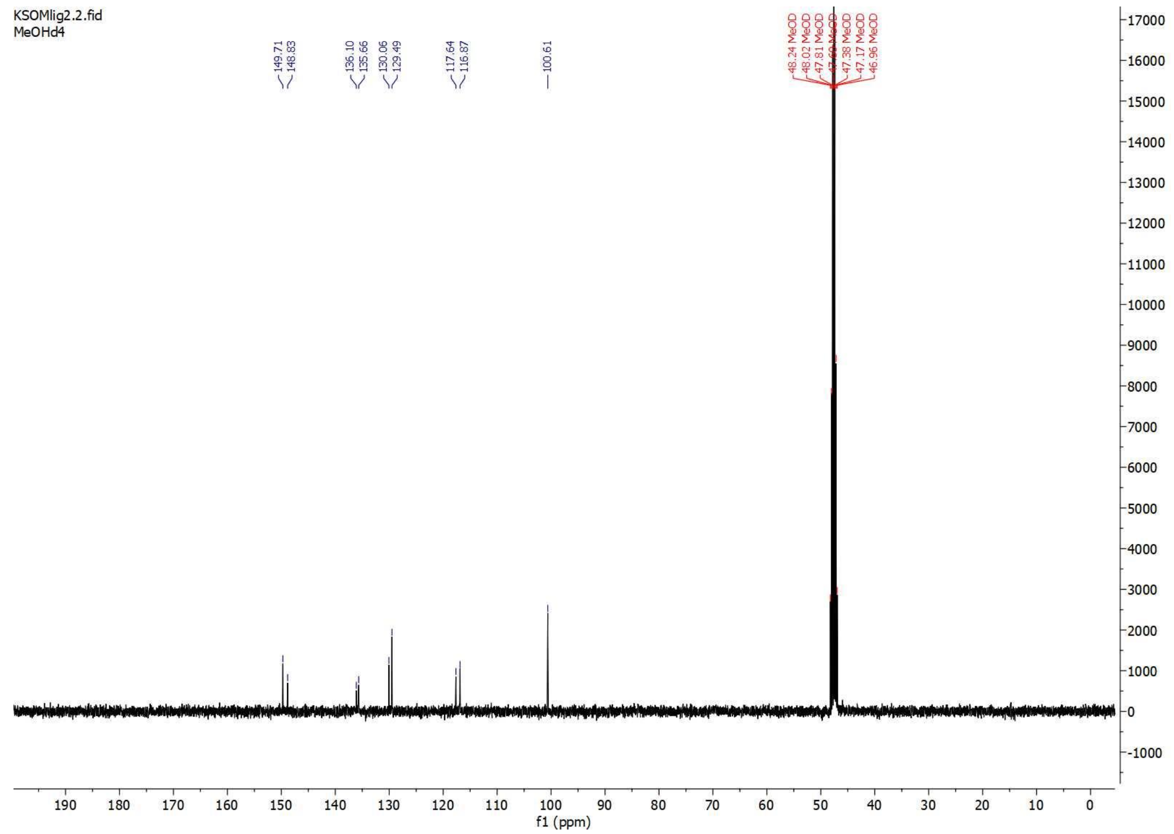

Figure S2. $^{13}C\{^{1}H\}$ NMR spectrum of the (iH)$_3$py ligand precursor in CD_3OD (5 mM).

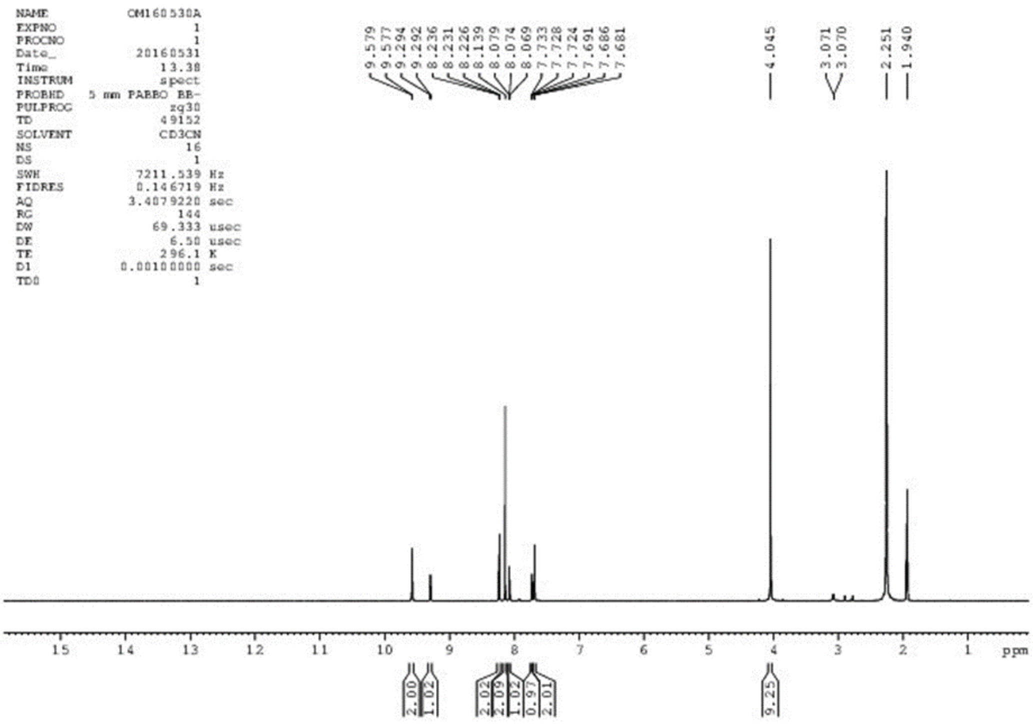

Figure S3. ^{1}H NMR spectrum of the $[(miH)_3py](PF_6)_3$ pre-ligand in CD_3CN (5 mM).

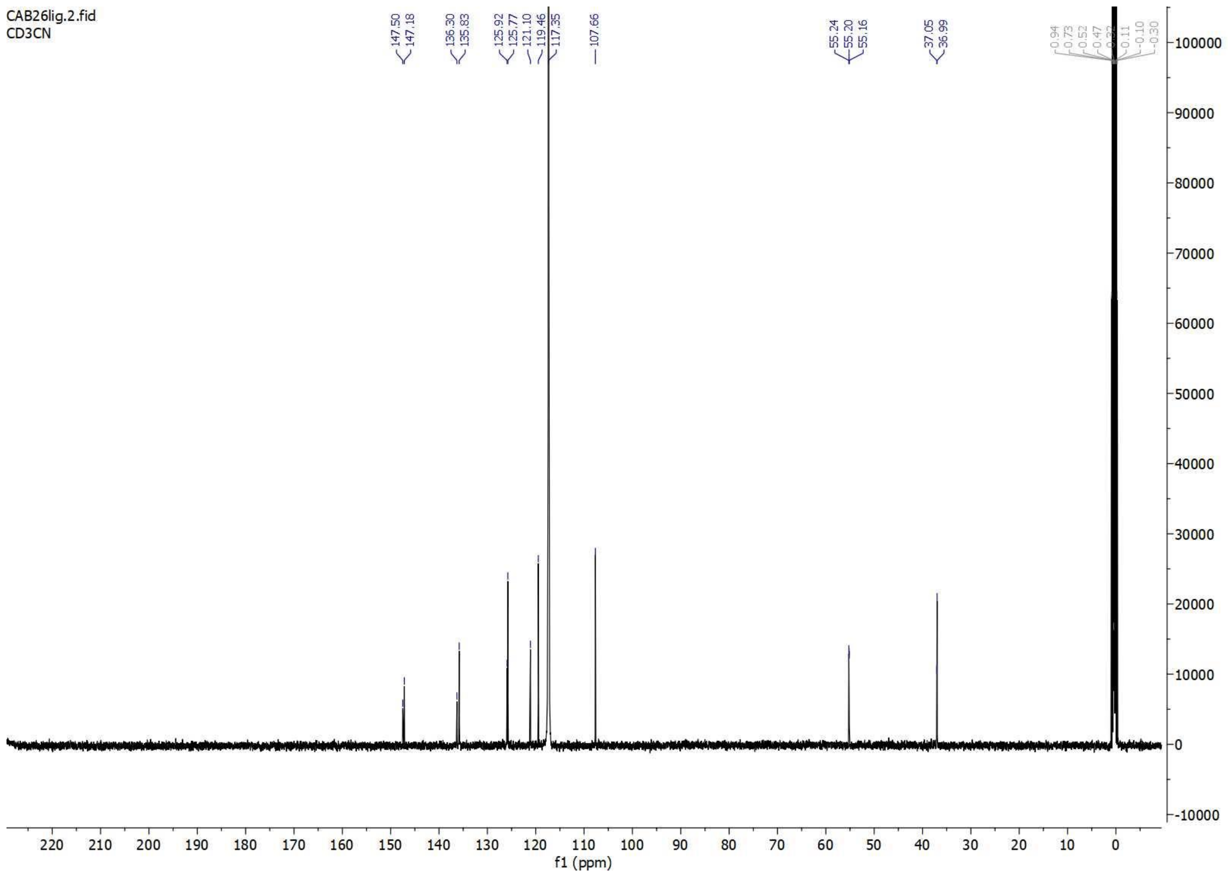

Figure S4. $^{13}C\{^{1}H\}$ NMR spectrum of the $[(miH)_3py](PF_6)_3$ pre-ligand in CD_3CN (5 mM).

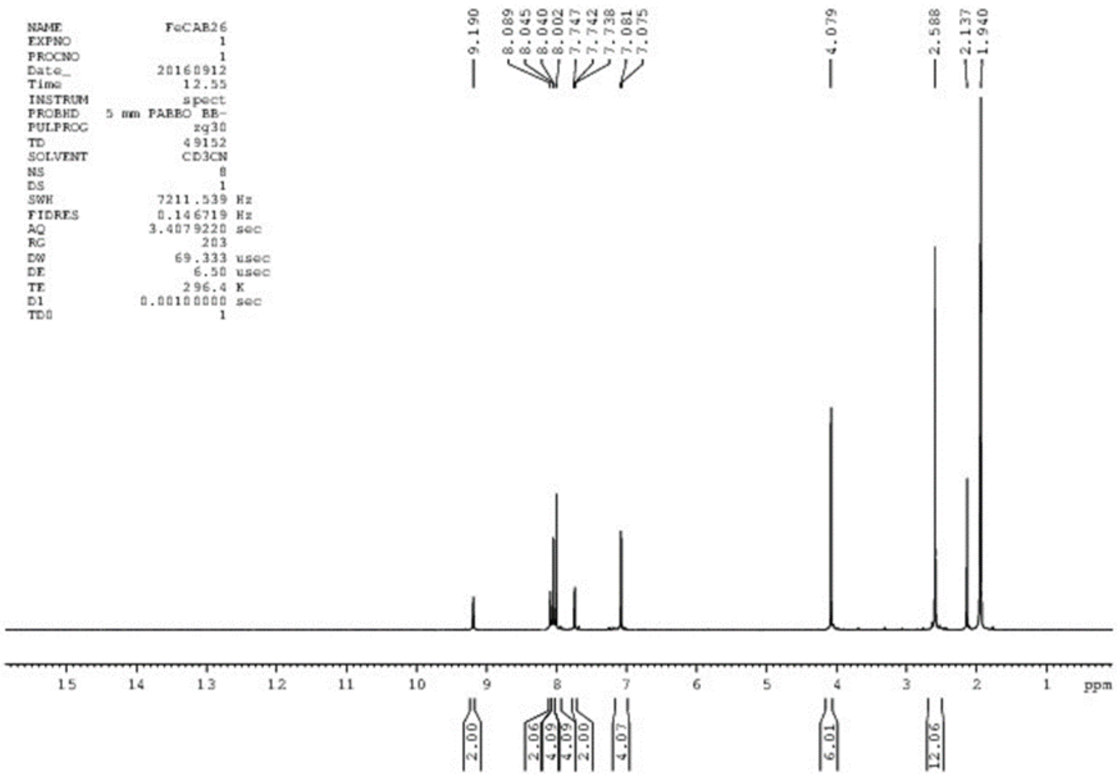

Figure S5. ^{1}H NMR spectrum of $[Fe(miHpbmi)_2](PF_6)_4$ in CD_3CN (20 mM).

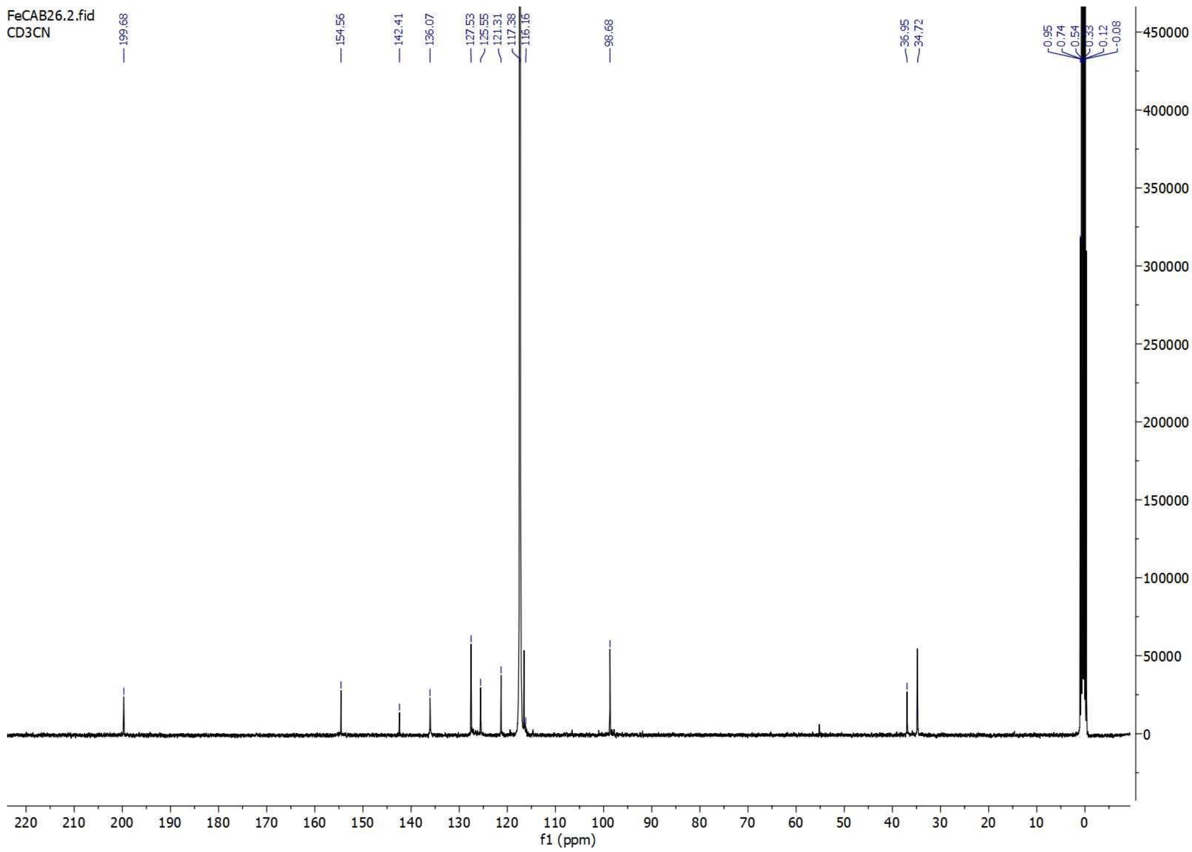

Figure S6. $^{13}C\{^{1}H\}$ NMR spectrum of $[Fe(miHpbmi)_2](PF_6)_4$ in CD_3CN (20 mM).

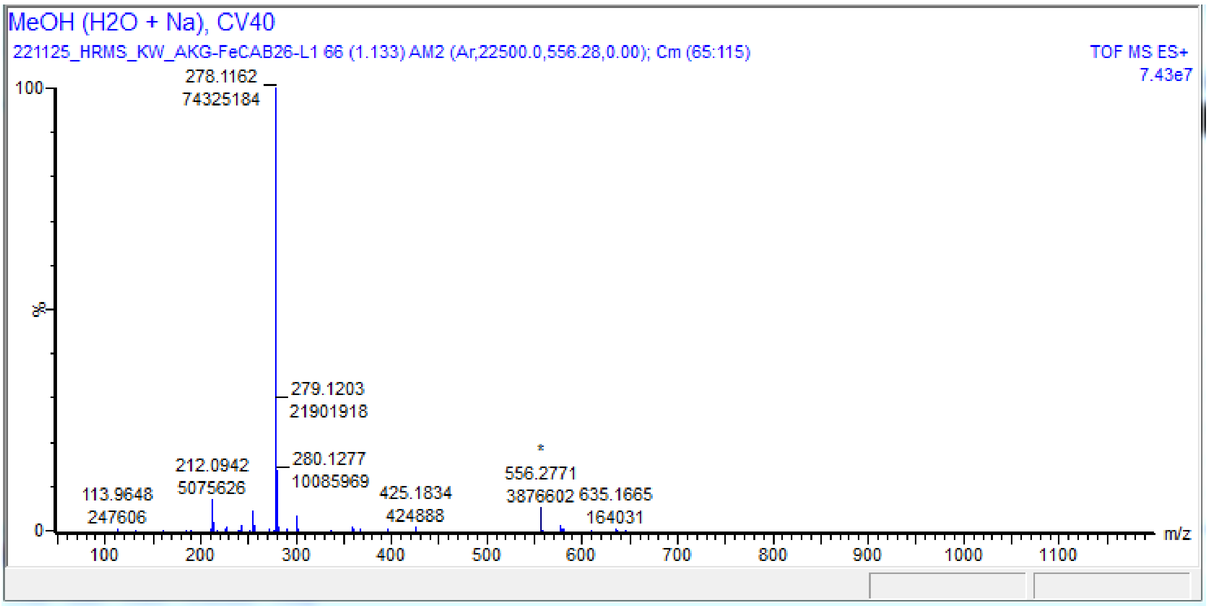

Single Mass Analysis

Tolerance = 2.0 mDa / DBE: min = -1.5, max = 100.0

Element prediction: Off

Number of isotope peaks used for i-FIT = 2

Monoisotopic Mass, Even Electron Ions

36 formula(e) evaluated with 1 results within limits (all results (up to 1000) for each mass)

Elements Used:

C: 1-50 H: 1-50 N: 1-10

Mass	Calc. Mass	mDa	PPM	DBE	Formula	i-FIT	i-FIT Norm	Fit Conf %	C	H	N
278.1162	278.1154	0.8	2.9	12.5	C14 H12 N7	297.2	n/a	n/a	14	12	7

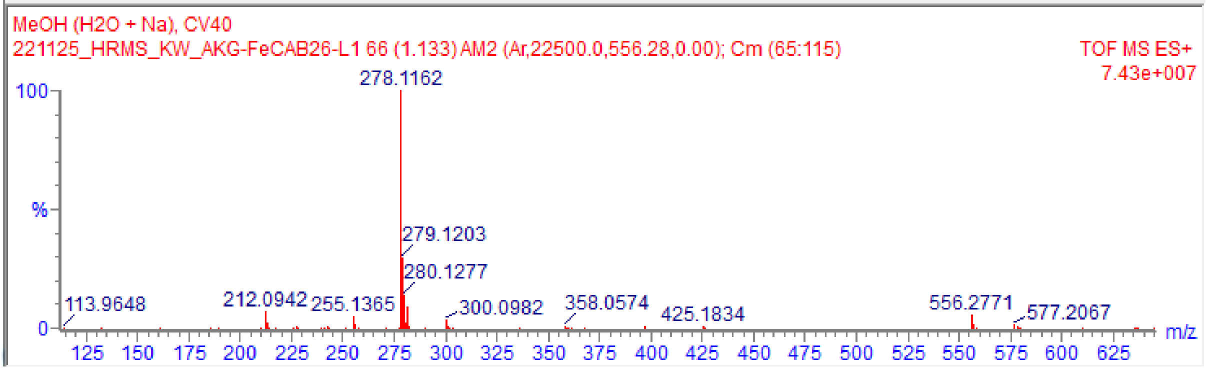

Figure S7: HRMS spectrum of (iH)$_3$py.

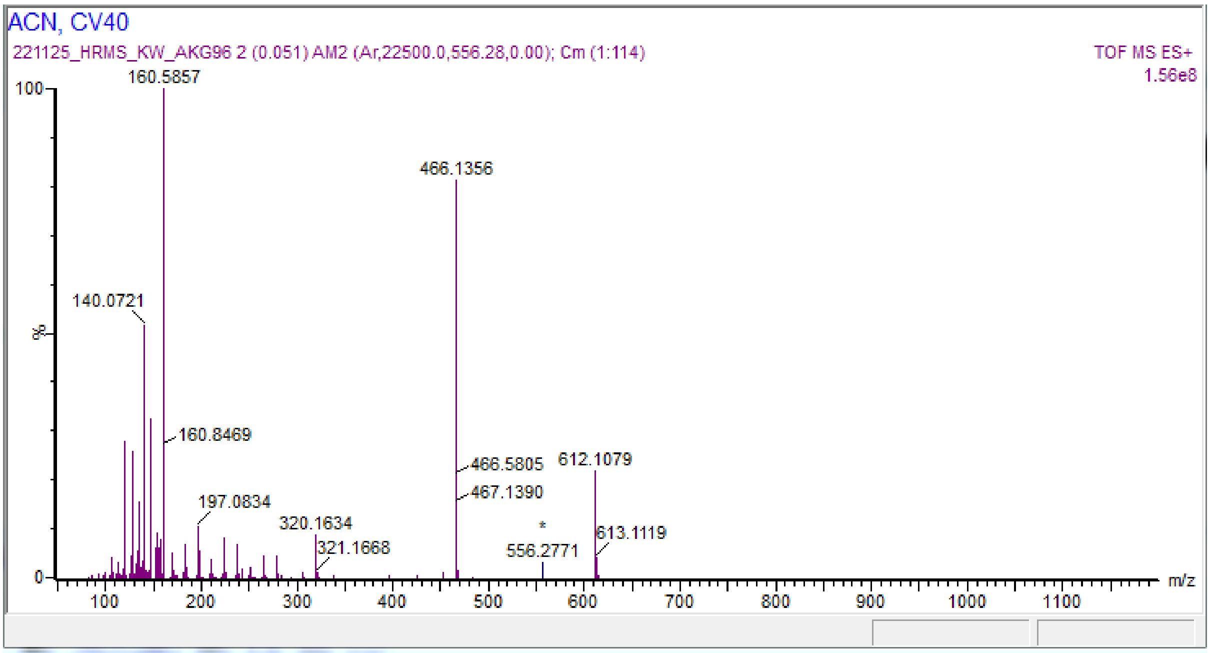

Single Mass Analysis
Tolerance = 2.0 mDa / DBE: min = -1.5, max = 100.0
Element prediction: Off
Number of isotope peaks used for i-FIT = 2
Monoisotopic Mass, Even Electron Ions
34 formula(e) evaluated with 1 results within limits (all results (up to 1000) for each mass)
Elements Used:
C: 1-50 H: 1-50 N: 1-10 F: 12-12 P: 2-2

Mass	Calc. Mass	mDa	PPM	DBE	Formula	i-FIT	i-FIT Norm	Fit Conf %	C	H
612.1079	612.1064	1.5	2.5	6.5	C17 H20 N7 F12 P2	60.8	n/a	n/a	17	20

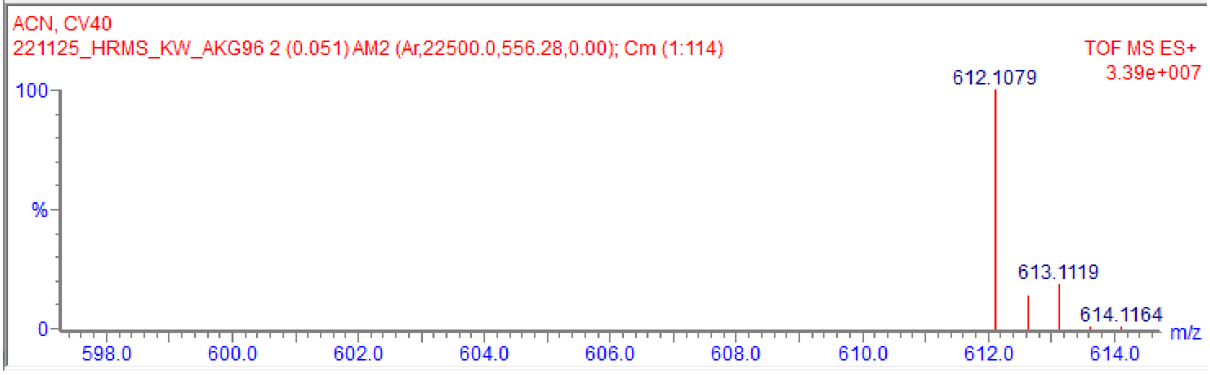

Figure S8: HRMS spectrum of $[(miH)_3py](PF_6)_3$.

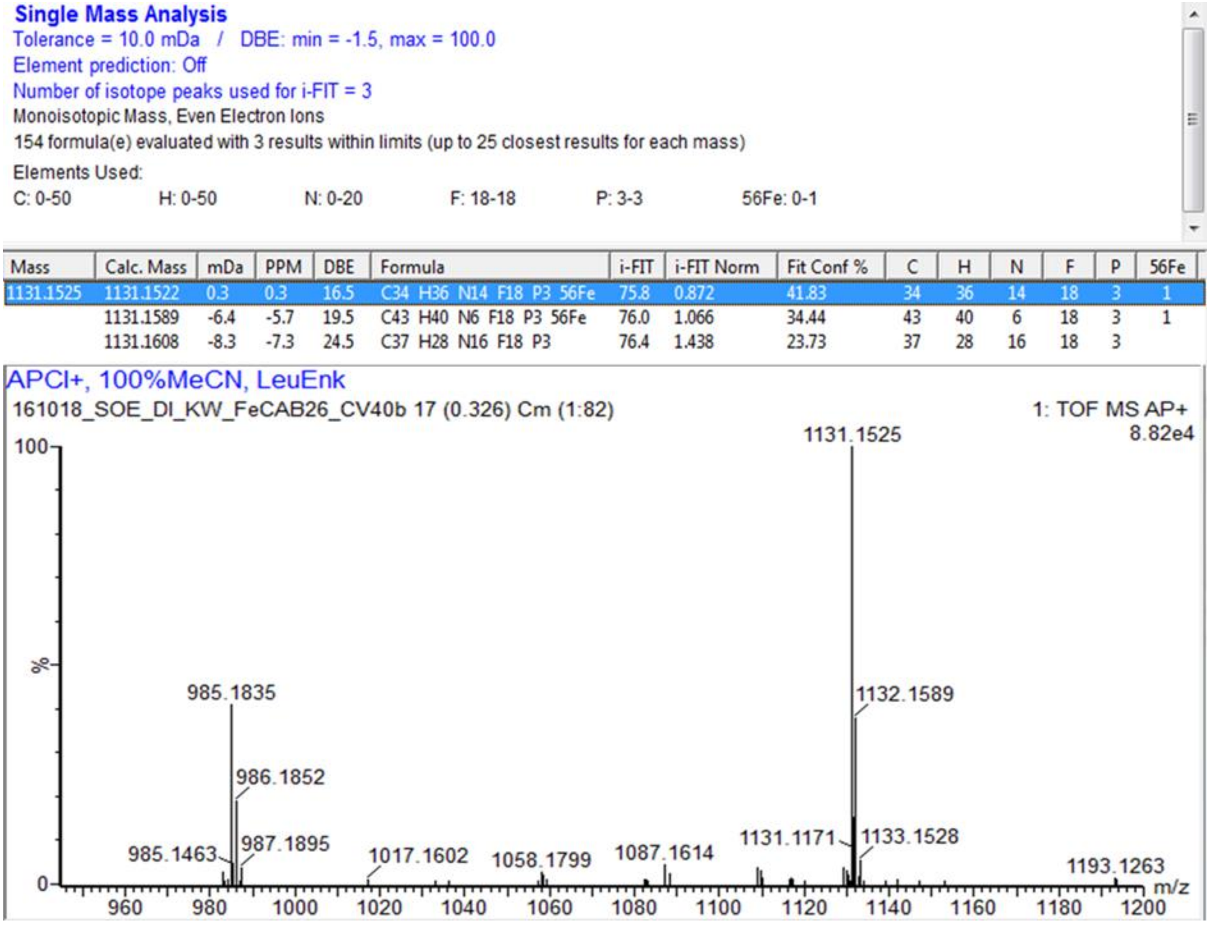

Figure S9. HR-MS spectrum of complex $[Fe(miHpbmi)_2](PF_6)_4$.

S2. Single Crystal X-ray diffraction

All SC-XRD measurements were performed using graphite-monochromatized Mo Kα radiation (λ = 0.71073 Å) using the Agilent Xcalibur Sapphire3 diffractometer high-brilliance IμS radiation source. Data collections were performed at 295 K for all the structures. The structure was solved by direct methods and refined by full-matrix least-squares techniques against F2 using all data (SHELXT, SHELXS).[S1,S2] All non-hydrogen atoms were refined with anisotropic displacement parameters if not stated otherwise. Absorption was corrected using multi-scan empirical absorption correction with spherical harmonics as implemented in the SCALE3 ABSPACK scaling algorithm.[S3] Hydrogen atoms were constrained in geometric positions to their parent atoms using OLEX2 software.[S4]

Table S1. Crystal data and structure refinement for $(iH)_3py$.

Identification code	$(iH)_3py$	
CCDC No.	2258761	
Empirical formula	$C_{14}H_{11}N_7$	
Formula weight	277.30	
Temperature	293 K	
Wavelength	0.71073 Å	
Crystal system	Monoclinic	
Space group	P 1 21/n 1	
Unit cell dimensions	a = 14.842(3) Å	a= 90°.
	b = 5.628(2) Å	b= 99.26(2)°.
	c = 17.889(5) Å	g = 90°.
Volume	1474.7(8) $Å^3$	
Z	4	
Density (calculated)	1.249 Mg/m^3	
Absorption coefficient	0.083 mm^{-1}	
F(000)	576	
Crystal size	0.38 x 0.3 x 0.26 mm^3	
Theta range for data collection	3.517 to 24.996°.	
Index ranges	-16<=h<=17, -6<=k<=5, -20<=l<=21	
Reflections collected	8957	
Independent reflections	2544 [R(int) = 0.0724]	
Completeness to theta = 24.996°	97.8 %	
Absorption correction	Semi-empirical from equivalents	
Max. and min. transmission	1.00000 and 0.33307	
Refinement method	Full-matrix least-squares on F^2	
Data / restraints / parameters	2544 / 0 / 190	
Goodness-of-fit on F^2	0.988	
Final R indices [I>2sigma(I)]	R1 = 0.0865, wR2 = 0.2250	
R indices (all data)	R1 = 0.1415, wR2 = 0.2719	
Extinction coefficient	n/a	
Largest diff. peak and hole	0.311 and -0.244 e.$Å^{-3}$	

Table S2. Crystal data and structure refinement for $[(miH)_3py](PF_6)_3$.

Identification code	$[(miH)_3py](PF_6)_3$	
CCDC No.	2258760	
Empirical formula	$C_{17}H_{20}F_{18}N_7P_3$	
Formula weight	757.31	
Temperature	293 K	
Wavelength	0.71073 Å	
Crystal system	Monoclinic	
Space group	P 1 21/c 1	
Unit cell dimensions	a = 8.5316(7) Å	a= 90°.
	b = 21.1207(14) Å	b= 93.792(6)°.
	c = 15.9934(10) Å	g = 90°.
Volume	2875.6(4) Å^3	
Z	4	
Density (calculated)	1.749 Mg/m^3	
Absorption coefficient	0.348 mm^{-1}	
F(000)	1512	
Crystal size	0.32 x 0.28 x 0.16 mm^3	
Theta range for data collection	3.389 to 24.998°.	
Index ranges	-9<=h<=10, -25<=k<=24, -19<=l<=19	
Reflections collected	32712	
Independent reflections	5038 [R(int) = 0.0778]	
Completeness to theta = 24.998°	99.7 %	
Absorption correction	Semi-empirical from equivalents	
Max. and min. transmission	1.00000 and 0.92197	
Refinement method	Full-matrix least-squares on F^2	
Data / restraints / parameters	5038 / 7 / 464	
Goodness-of-fit on F^2	1.033	
Final R indices [I>2sigma(I)]	R1 = 0.0917, wR2 = 0.2051	
R indices (all data)	R1 = 0.1354, wR2 = 0.2325	
Extinction coefficient	n/a	
Largest diff. peak and hole	0.432 and -0.355 e.Å	

Table S3. Crystal data and structure refinement for [Fe(miHpbmi)$_2$](PF$_6$)$_4$.

Identification code	[Fe(miHpbmi)$_2$](PF$_6$)$_4$	
CCDC No.	2218451	
Empirical formula	$C_{34}H_{36}F_{24}FeN_{14}P_4$	
Formula weight	1276.50	
Temperature	293 K	
Wavelength	0.71073 Å	
Crystal system	Monoclinic	
Space group	C 1 2/c 1	
Unit cell dimensions	a = 14.248(2) Å	a= 90°.
	b = 30.315(2) Å	b= 117.934(15)°.
	c = 13.1520(10) Å	g = 90°.
Volume	5018.8(11) Å^3	
Z	4	
Density (calculated)	1.689 Mg/m^3	
Absorption coefficient	0.562 mm^{-1}	
F(000)	2560	
Crystal size	0.15 x 0.15 x 0.15 mm^3	
Theta range for data collection	3.168 to 28.988°.	
Index ranges	-18<=h<=18, -40<=k<=40, -15<=l<=17	
Reflections collected	8887	
Independent reflections	8887 [R(int) = ?]	
Completeness to theta = 25.242°	99.8 %	
Absorption correction	Semi-empirical from equivalents	
Max. and min. transmission	1.00000 and 0.90563	
Refinement method	Full-matrix least-squares on F^2	
Data / restraints / parameters	8887 / 146 / 409	
Goodness-of-fit on F^2	1.026	
Final R indices [I>2sigma(I)]	R1 = 0.0688, wR2 = 0.1667	
R indices (all data)	R1 = 0.1151, wR2 = 0.1938	
Extinction coefficient	n/a	
Largest diff. peak and hole	0.937 and -0.555 e.Å^{-3}	

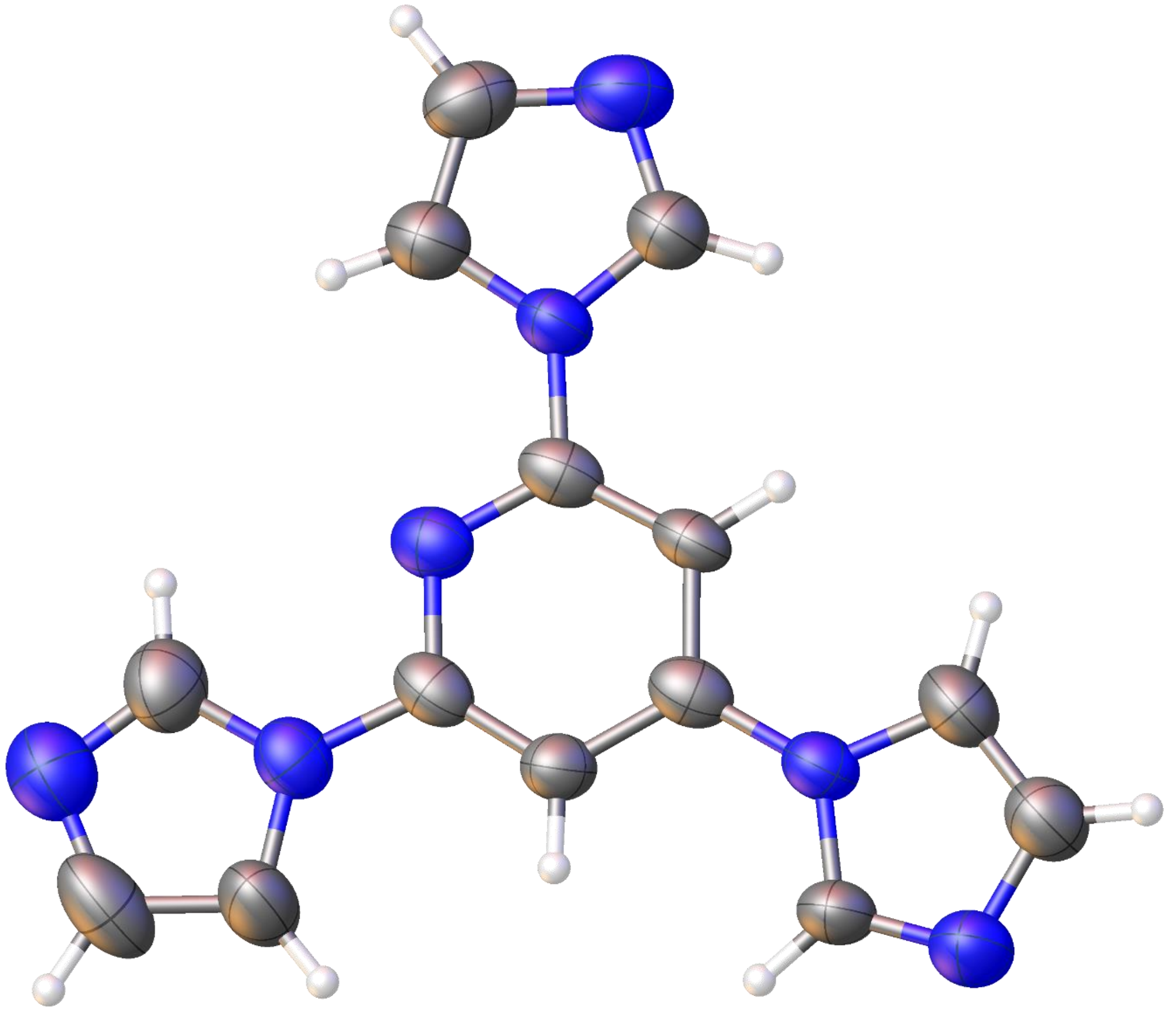

Figure S10: Molecular structure of (iH)$_3$py as determined by SC-XRD. Thermal ellipsoids are shown at 30% probability. Displayed atoms are C-black, N-blue, H-white.

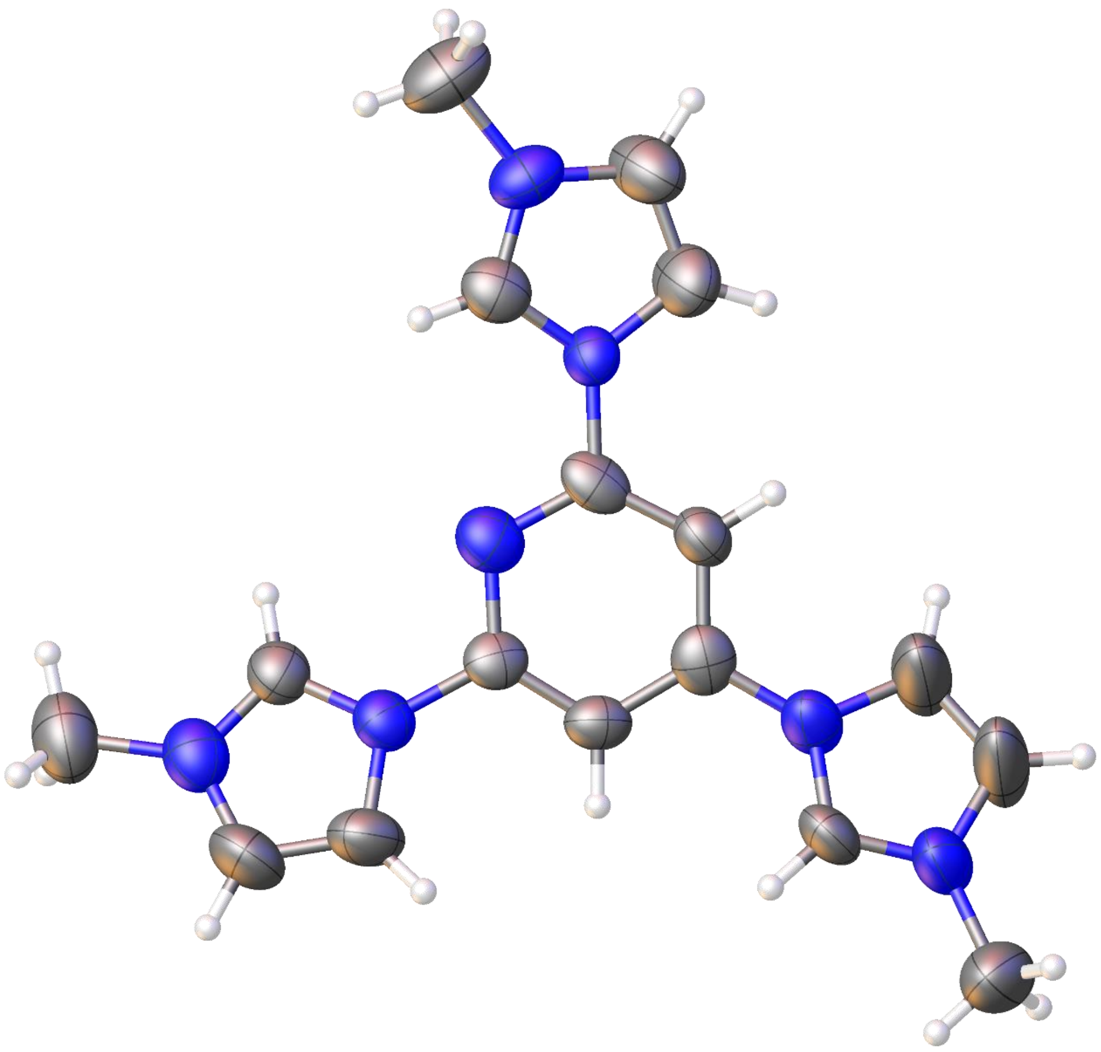

Figure S11: Molecular structure of $[(miH)_3py](PF_6)_3$ as determined by SC-XRD. Thermal ellipsoids are shown at 30% probability. Counterions and solvents are omitted for clarity. The displayed atoms are carbon-black; nitrogen-blue and hydrogen-white. The PF_6 counter ions were omitted for clarity.

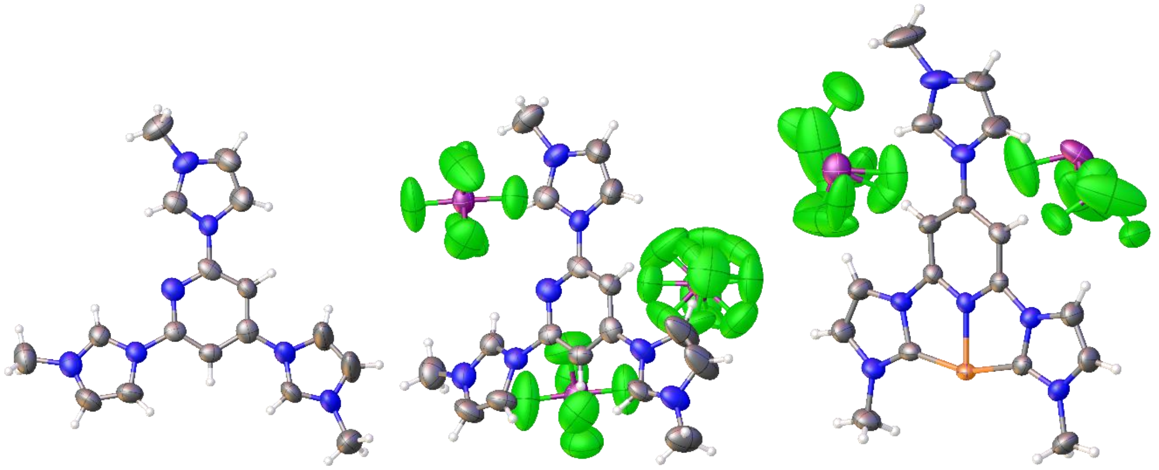

Figure S12: Asymmetric unit of (iH)$_3$py, [(miH)$_3$py](PF$_6$)$_3$ and [Fe(miHpbmi)$_2$](PF$_6$)$_4$. carbon-black; nitrogen-blue; phosphorus-purple; florine-green; hydrogen-white.

Table S4: Selected bond lengths and bond angles of the compound (iH)$_3$py, [(miH)$_3$py](PF$_6$)$_3$, and [Fe(miHpbmi)$_2$](PF$_6$)$_4$.

**Compound**	**Bond lengths (Å)**	**Bond angles (^O)**
(iH)$_3$py	C(1)-N(1): 1.330(5)	C(2)-C(1)-N(2): 121.1(4)
	C(1)-N(2): 1.386(5)	N(1)-C(1)-C(2): 123.6(4)
	C(3)-N(6): 1.397(5)	N(1)-C(1)-N(2): 115.2(4)
	C(15)-N(1): 1.351(5)	C(4)-C(3)-N(6): 120.1(3)
	C(15)-N(4): 1.415(5)	N(6)-C(3)-C(2): 120.9(4)
	C(6)-N(2): 1.373(5)	C(4)-C(15)-N(4): 121.5(4)
	C(7)-N(3): 1.393(6)	N(1)-C(15)-C(4): 123.9(4)
	C(8)-N(3): 1.331(5)	N(1)-C(15)-N(4): 114.6(4)
	C(9)-N(10): 1.295(6)	C(7)-C(6)-N(2): 107.2(4
	C(9)-N(4): 1.341(6)	C(6)-C(7)-N(3): 109.6(4)
	N(10)-C(5): 1.338(6)	N(3)-C(7)-H(7): 125.2
	C(11)-N(4): 1.366(5)	N(3)-C(8)-N(2): 112.4(4)
	C(12)-N(6): 1.353(4)	N(10)-C(9)-N(4): 113.0(4)
	C(12)-N(7): 1.300(5)	C(9)-N(10)-C(5): 104.9(5)
	C(13)-N(6): 1.375(5)	N(4)-C(11)-C(5): 103.7(5)
		N(7)-C(12)-N(6): 112.9(4)
		C(14)-C(13)-N(6): 105.6(4)
		C(13)-C(14)-N(7): 111.5(4)
		C(1)-N(1)-C(15): 117.0(4)

		C(6)-N(2)-C(1): 126.0(4)<br>C(8)-N(2)-C(1): 127.6(4)<br>C(8)-N(2)-C(6): 106.3(3)<br>C(8)-N(3)-C(7): 104.4(4)<br>C(9)-N(4)-C(15): 124.2(4)<br>C(9)-N(4)-C(11): 107.0(4)<br>C(11)-N(4)-C(15): 128.7(4)<br>N(10)-C(5)-C(11): 111.4(5)<br>C(12)-N(6)-C(3): 127.2(3)<br>C(12)-N(6)-C(13): 105.7(4)<br>C(13)-N(6)-C(3): 127.1(3)<br>C(12)-N(7)-C(14): 104.3(4)
$[(miH)_3py](PF_6)_3$	C(8)-N(6): 1.404(7)<br>C(6)-N(1): 1.349(6)<br>C(6)-N(4): 1.419(6)<br>C(5)-N(1): 1.353(6)<br>C(13)-N(4): 1.379(7)<br>C(12)-N(5): 1.393(7)<br>C(10)-N(4): 1.333(6)<br>C(11)-N(5): 1.487(7)<br>C(4)-N(2): 1.384(7)<br>C(3)-N(3): 1.348(8)<br>C(2)-N(2): 1.337(6)<br>C(2)-N(3): 1.325(7)<br>C(1)-N(3): 1.511(7)<br>C(14)-N(6): 1.388(7)<br>C(15)-N(7): 1.363(8)<br>C(17)-N(6): 1.320(7)<br>C(17)-N(7): 1.300(7)<br>C(16)-N(7): 1.481(8)	C(7)-C(8)-N(6): 120.4(5)<br>C(7)-C(6)-N(1): 124.6(5)<br>C(7)-C(6)-N(4): 118.6(4)<br>N(1)-C(6)-N(4): 116.7(5)<br>C(9)-C(5)-N(1): 124.1(5)<br>C(9)-C(5)-N(2): 120.2(5)<br>N(1)-C(5)-N(2): 115.7(4)<br>C(12)-C(13)-N(4): 107.8(5)<br>C(13)-C(12)-N(5): 106.8(5)<br>N(5)-C(10)-N(4): 108.6(5)<br>C(3)-C(4)-N(2): 106.1(6)<br>N(3)-C(3)-C(4): 107.8(5)<br>N(3)-C(2)-N(2): 107.9(5)<br>C(14)-C(15)-N(7): 108.1(6)<br>N(7)-C(17)-N(6): 110.2(5)
**$[Fe(miHpbmi)_2](PF_6)_4$**	C(13)-N(1): 1.468(7)<br>C(11)-N(1): 1.390(7)<br>C(10)-N(2): 1.385(7)<br>C(12)-N(1): 1.332(6)<br>C(12)-N(2): 1.375(6)<br>C(12)-Fe(1): 1.958(5)<br>C(17)-N(5): 1.456(7)<br>C(16)-N(4): 1.378(6)	N(1)-C(12)-Fe(1): 143.2(4)<br>N(2)-C(12)-Fe(1): 113.0(3<br>N(4)-C(16)-Fe(1): 113.6(4)<br>N(5)-C(16)-Fe(1): 142.9(4)<br>C(9)-N(3)-Fe(1): 121.4(3)<br>C(7)-N(3)-Fe(1): 120.6(3)<br>C(10)-C(11)-N(1): 107.8(5)<br>C(11)-C(10)-N(2): 105.8(5)

	C(16)-N(5): 1.337(7)<br>C(16)-Fe(1): 1.961(5)<br>C(15)-N(5): 1.386(7)<br>C(14)-N(4): 1.370(7)<br>C(9)-C(8): 1.376(7)<br>C(9)-N(3): 1.336(6)<br>C(9)-N(4): 1.392(6)<br>C(5)-N(6): 1.421(6)<br>C(7)-N(2): 1.385(6)<br>C(7)-N(3): 1.349(6)<br>C(1)-N(7): 1.479(9)<br>C(4)-N(6): 1.338(7)<br>C(4)-N(7): 1.309(7)<br>C(3)-N(6): 1.363(7)<br>C(2)-N(7): 1.351(9)<br>N(3)-Fe(1): 1.906(4)	N(1)-C(12)-N(2): 103.7(4)<br>N(5)-C(16)-N(4): 103.4(4)<br>C(14)-C(15)-N(5): 108.5(5)<br>C(8)-C(9)-N(4): 126.4(5)<br>N(3)-C(9)-C(8): 124.1(4)<br>N(3)-C(9)-N(4): 109.5(4)<br>C(8)-C(5)-N(6): 118.9(4)<br>N(3)-C(7)-N(2): 109.3(4)<br>C(11)-N(1)-C(13): 123.2(5)<br>C(12)-N(1)-C(13): 125.4(5)<br>C(12)-N(1)-C(11): 111.4(5)<br>C(12)-N(2)-C(10): 111.4(5)<br>C(12)-N(2)-C(7): 117.6(4)<br>C(7)-N(2)-C(10): 130.9(5)<br>C(9)-N(3)-C(7): 117.9(4)<br>C(16)-N(4)-C(9): 116.7(4)<br>C(14)-N(4)-C(16): 112.0(4)<br>C(14)-N(4)-C(9): 131.1(5)<br>C(16)-N(5)-C(17): 124.5(5)<br>C(16)-N(5)-C(15): 110.6(4)<br>C(15)-N(5)-C(17): 124.9(5)<br>C(4)-N(6)-C(5): 125.4(4)<br>C(4)-N(6)-C(3): 106.7(5)<br>C(3)-N(6)-C(5): 127.8(5)<br>C(4)-N(7)-C(1): 125.2(7)<br>C(4)-N(7)-C(2): 108.7(5)<br>C(2)-N(7)-C(1): 126.1(6)

S3. Mößbauer spectroscopy

Mössbauer measurements were carried out in an Oxford Instrument flow cryostat at 295 K and 85 K, using a 57CoRh source held at room temperature. The studied powder materials were mixed with inert BN, pressed and formed as disc absorbers with a concentration of about 52 mg/cm2 of studied substances. Calibration spectra were recorded from a natural iron metal foil held at 295 K. The resulting spectra were analysed using a least square Mössbauer fitting program.

S4. Electrochemistry and spectroelectrochemsitry

Electrochemical and spectro-electrochemical measurements were carried out in a standard three electrode setup consisting of a working (1 mm dia., glassy carbon, CH Instruments), counter (platinum rod in a separate compartment) and reference electrode (0.01 M Ag^+/Ag). Spectroscopic grade acetonitrile dried for 48 h over 3 Å activated molecular sieves was used as solvent, together with 0.1 M tetrabutylammonium hexafluorophosphate (electrochemical grade, Sigma) dried for 24 h under vacuum at 80ºC as supporting electrolyte. Sample solutions were deaerated by purging with solvent saturated Ar. Cyclic voltammograms were record at 0.05 V/s, and differential pulse voltammograms with step potential: 5 mV, modulation amplitude: 25 mV, modulation time: 0.05 s, interval time: 0.1 s. UV-Vis spectroelectrochemistry was carried out during controlled potential electrolysis in the same cell by switching the working electrode to a platinum mesh electrode placed in the 1 mm optical path. An Autolab potentiostat (PGSTAT302) was used to control the three-electrode setup using the GPES 4.9 software, and an Agilent 8453 diode array spectrophotometer was used to record the spectral traces.

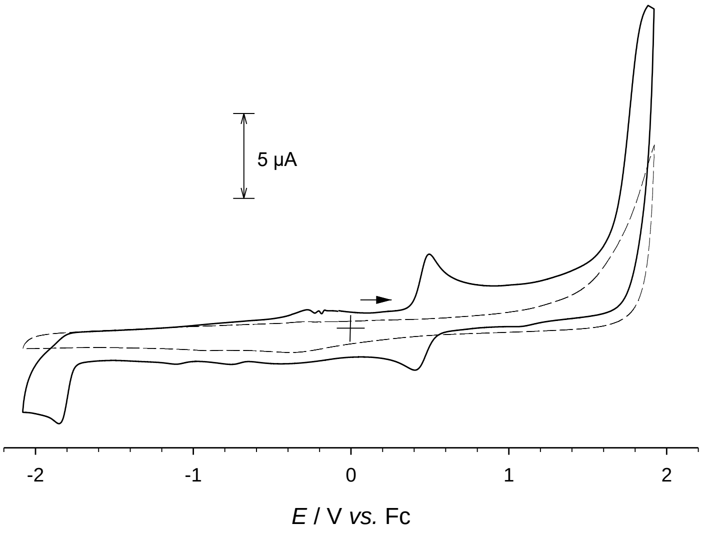

Figure S13. Cyclic voltammogram (50 mVs^{-1}) of $[Fe(miHpbmi)_2](PF_6)_4$ (1 mM) in acetonitrile with 0.1 M $TBAPF_6$ (—) and electrolyte background (---)

S5. Steady State Absorption Spectroscopy

Steady-state absorption measurements were performed in a Perkin Elmer Lambda 1050 Spectrophotometer. The complex was weighed and dissolved in filtered acetonitrile collected from a dry solvent dispenser (Innovative technology, PS-micro). In volumetric flasks, a dilution series was prepared by taking variable amounts of the stock solution and transferring it with a graded pipette, see Figure S14. Absorbance of all prepared concentrations was measured in a standard quartz-glass cuvette of path length 1 mm (Hellma – Optical Special Glass). For reference, the same cuvette with pure solvent was measured. The extinction coefficient was evaluated where appropriate by performing a linear fit to the absorbance as a function of concentration for each wavelength (see Figure S15) after the background had been corrected.

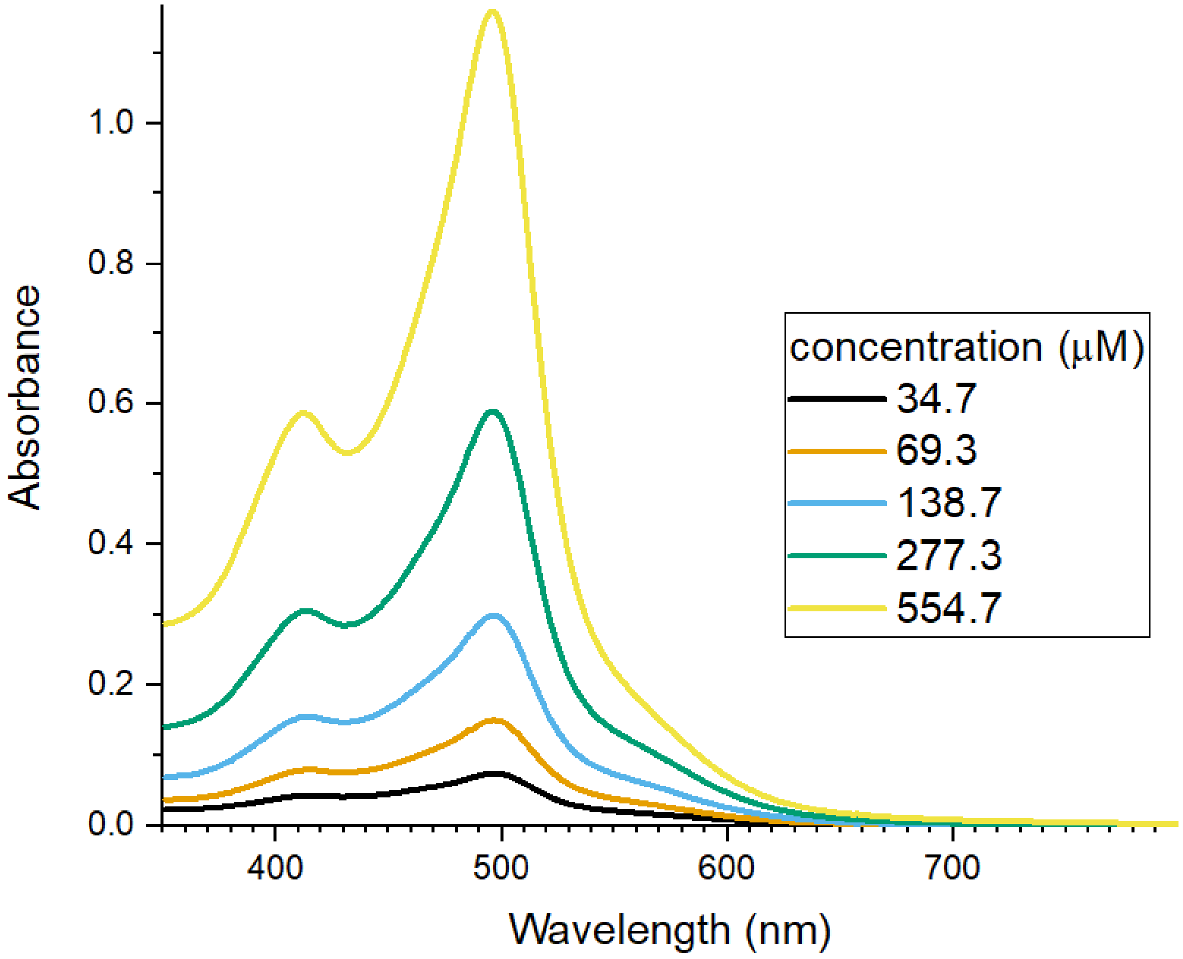

Figure S14. Absorption spectra of the dilution series of five different samples of $[Fe(miHpbmi)_2](PF_6)_4$ in dried acetonitrile used for the extinction calculations.

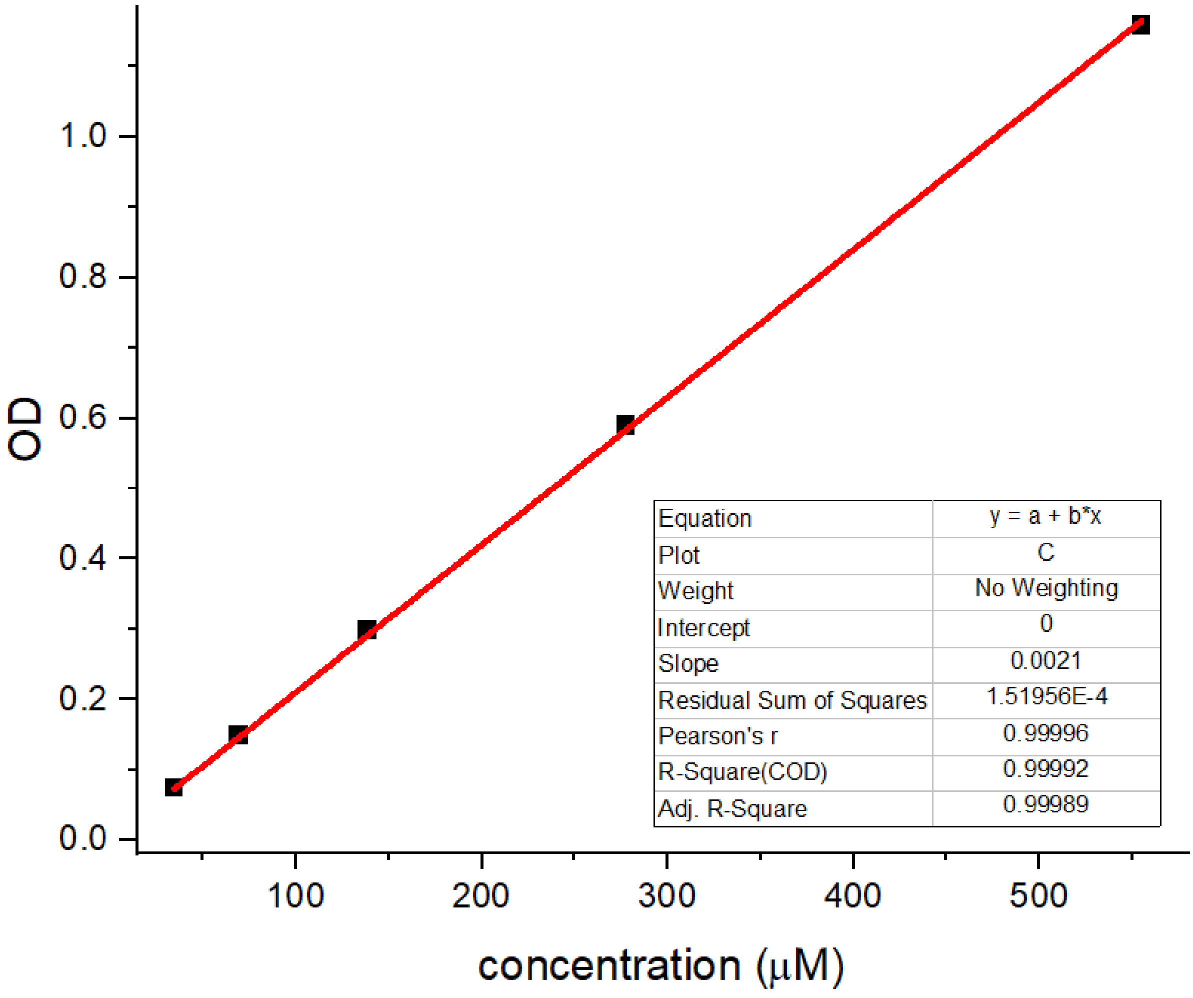

Figure S15. Linear fit of absorbance versus concentration of $[Fe(miHpbmi)_2](PF_6)_4$ at 495nm obtained from steady state absorption measurement.

S6. Transient absorption spectroscopy

Transient absorption (TA) spectroscopy was performed using an in-house build setup. The basis of this setup is a Spitfire Pro XP (Spectra Physics) laser amplifier system that produces ~80 fs pulses at a central wavelength of 796 nm at 1 kHz repetition rate. The amplifier output is divided into two parts that each pump a collinear optical parametric amplifier (TOPAS-C, Light Conversion). One of the TOPAS generates the pump beam (wavelength roughly set to

the absorption maximum of each sample, here ~500 nm), while the other one generates a NIR beam (1350 nm) that is focused onto a 5 mm CaF_2 crystal to generate a supercontinuum probe beam. The delay between the pump and probe beams is introduced by a computer-controlled delay stage (Aerotech) placed in the probe beam's path. After supercontinuum generation the probe pulses are split into two parts: the former being focused to ~100 µm spot size and overlapping with the pump pulse in the sample volume, and the latter serving as a reference. After passing the sample the probe beam is collimated again and relayed onto the entrance slit of a prism spectrograph. The reference beam is directly relayed on the said spectrograph. Both beams are then dispersed onto a double photodiode array, each holding 512 elements (Pascher Instruments). The intensity of excitation pulses was set to roughly 1 mW. Mutual polarization between pump and probe beams was set to the magic angle (54.7°) by placing a Berek compensator in the pump beam.

A solution of $[Fe(miHpbmi)_2](PF_6)_4$ in filtered acetonitrile was filled in a 1 mm optical path length cuvette (Hellma – Optical Special Glass) and measurements were performed at room temperature. The measured samples were translated after each scan to avoid photodegradation. To check for the stability of each sample steady-state absorption spectra were measured before and after TA experiments. Before analysis, the measured data were corrected for group velocity dispersion (GVD – "chirp") using the KiMoPack software.[S5] Data were fitted by using the KiMoPack global analysis software, not assuming any model only fitting a sum of exponential decay components convoluted with a rise component determined by the instrument response function ~90 fs.

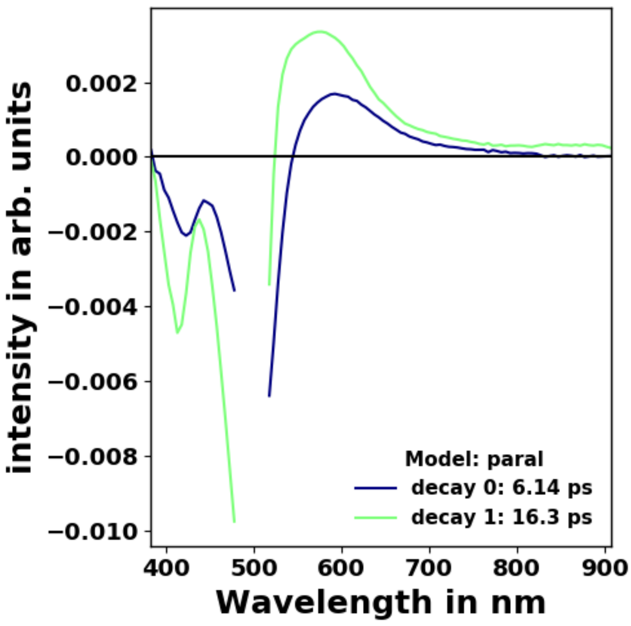

Figure S16. Decay associated spectra resulting from a global fit of the transient absorption data cut at 0.5 ps, corrected for background, chirp and cut to avoid excitation scatter.

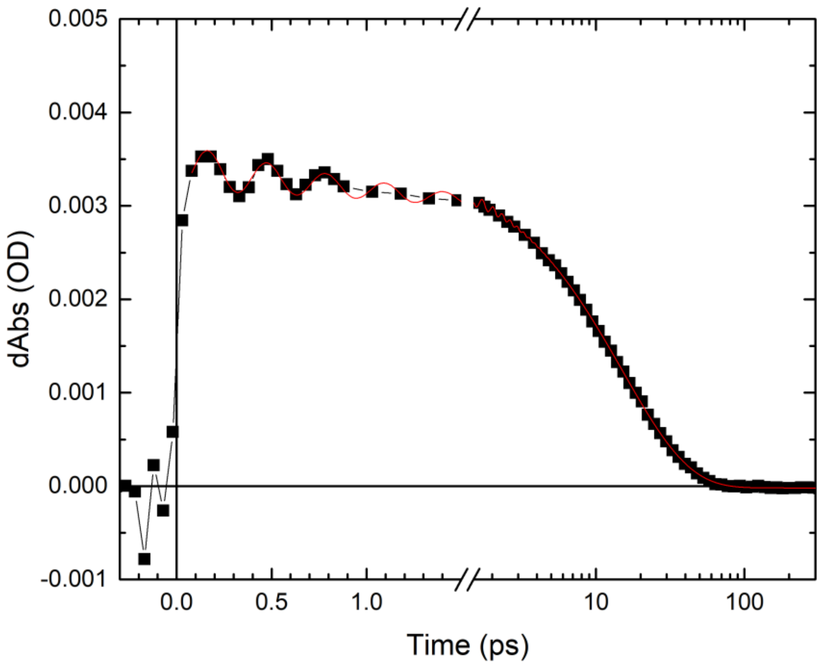

Figure S17. Transient absorption kinetic at 550 nm, fitted with a damped cosine function added to two decaying exponential functions.

TA data was also collected in the solvents dimethyl sulfoxide (DMSO), water (H_2O) and a mixture of acetonitrile (MeCN) and tetrahydrofuran (THF). The TA spectra are shown in Figures S18-S20. The dynamics in all solvents are similar within the signal to noise, see the kinetics at 600 nm plotted in Figure S21. Decay associate spectra resulting from the global fits to the data are presented in Figure S22. Also the oscillations are similar in all solvents, see Figure S23, with only slight variations in the fitted oscillatory parameters, see Table S5.

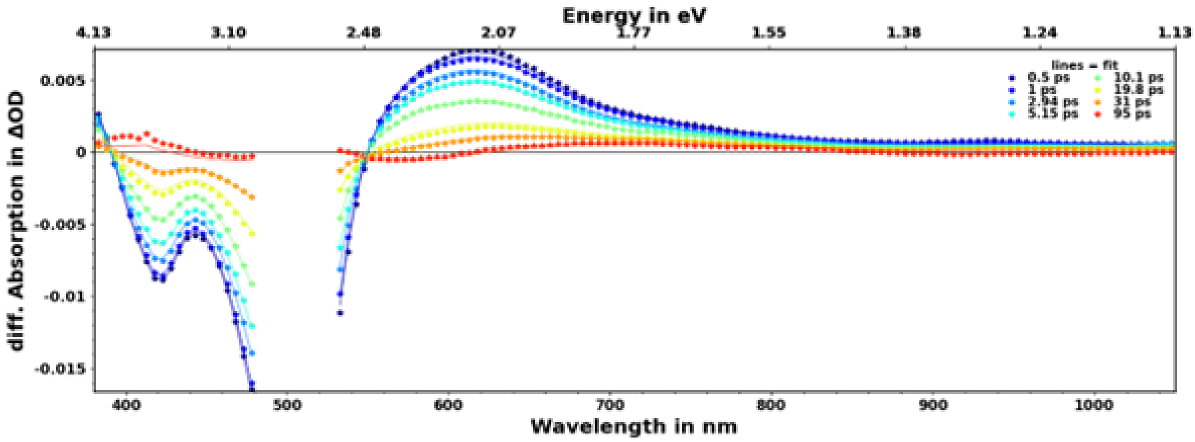

Figure S18. Transient absorption spectra of [Fe(miHpbmi)$_2$](PF$_6$)$_4$ in dimethyl sulfoxide after excitation at 500 nm, at selected delay times. Spectra have been chirp- and background-corrected, and also cut to remove excitation scatter.

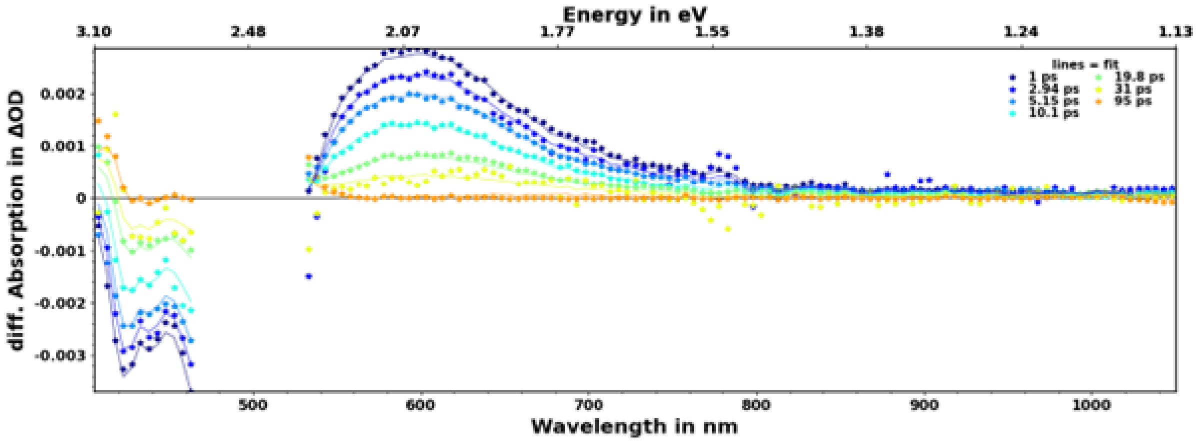

Figure S19. Transient absorption spectra of [Fe(miHpbmi)$_2$](PF$_6$)$_4$ in water after excitation at 500 nm, at selected delay times. Spectra have been chirp- and background-corrected, and also cut to remove excitation scatter.

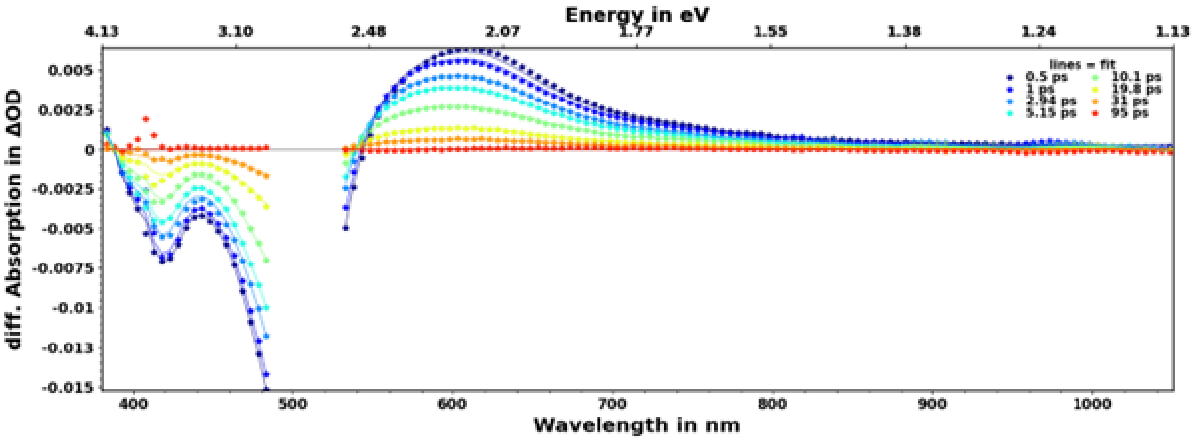

Figure S20. Transient absorption spectra of $[Fe(miHpbmi)_2](PF_6)_4$ in acetonitrile and tetrahydrofuran after excitation at 500 nm, at selected delay times. Spectra have been chirp- and background-corrected, and also cut to remove excitation scatter.

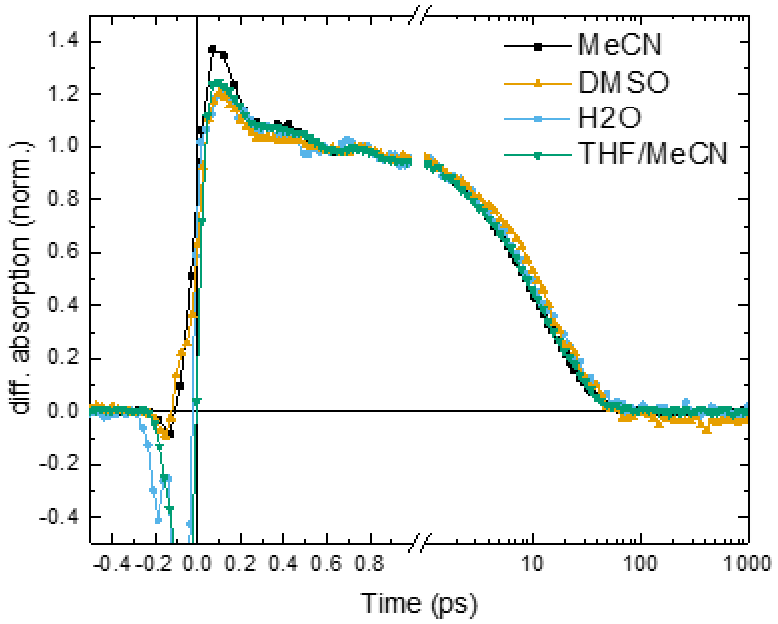

Figure S21. Transient absorption kinetics at 600 nm of $[Fe(miHpbmi)_2](PF_6)_4$ in different solvents after excitation at 500 nm. Data have been chirp- and background-corrected.

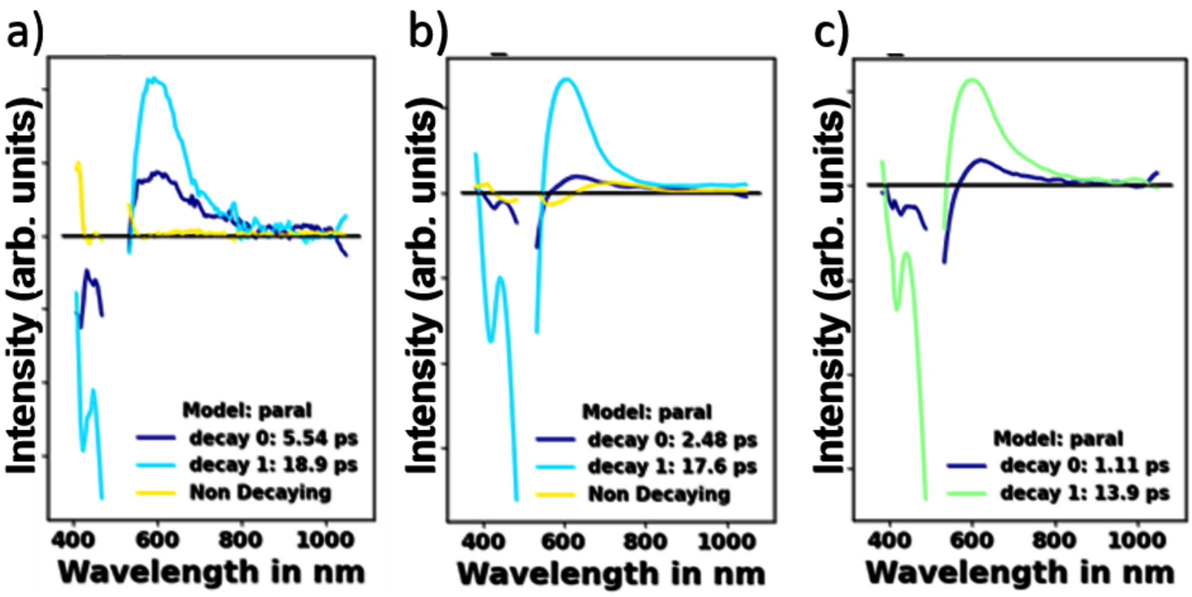

Figure S22. Decay associated spectra resulting from a global fits of the transient absorption data measured in a) H_2O, b) DMSO and c) 50/50% mixture of THF and MeCN, cut at 0.5 ps, corrected for background, chirp and cut to avoid excitation scatter.

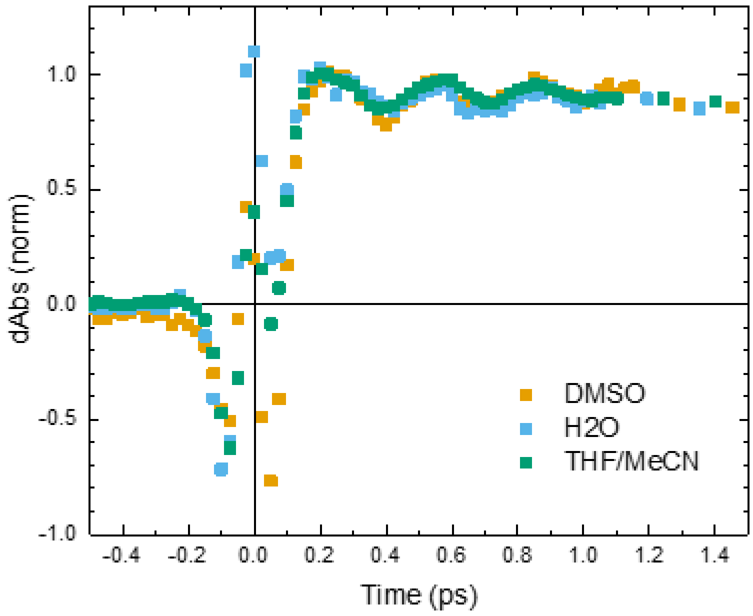

Figure S23. Transient absorption kinetics at 560 nm of $[Fe(miHpbmi)_2](PF_6)_4$ in different solvents after excitation at 500 nm. Data have been chirp- and background-corrected.

Table S5. Summary of the oscillations fitted for the transient absorption kinetic at 560 nm of $[Fe(miHpbmi)_2](PF_6)_4$ in different solvents. The solvent polarity is also included in the table.

Solvent	*Period time (fs)*	*Stretching frequency* $(2\pi ps^{-1})$	*Damping time (ps)*	*Viscosity (cP)*
MeCN[1]	*310*	*20*	*1.1*	*0.38*[2]
H2O	*340*	*19*	*0.7*	*1.0*
DMSO	*290*	*22*	*0.7*	*2.24*
THF+MeCN	*320*	*20*	*0.8*	*0.39*[3]

[1]*Kinetic at 550 nm fitted, see main manuscript.*

[2]*At 15°C.*

[3] Estimated for a 50:50 mixture from reference S6.

S7. Quantum Chemistry

Density Functional Theory (DFT) and Time-Dependent DFT (TD-DFT) calculations were performed by using the program Gaussian 09[S7] using the B3LYP*[S8] level of theory with the basis set 6-311G(d)[S9,S10], modelled in an empirical solvent model of acetonitrile. Kohn-Sham orbitals and spin density contours were visualized in the program GaussView.

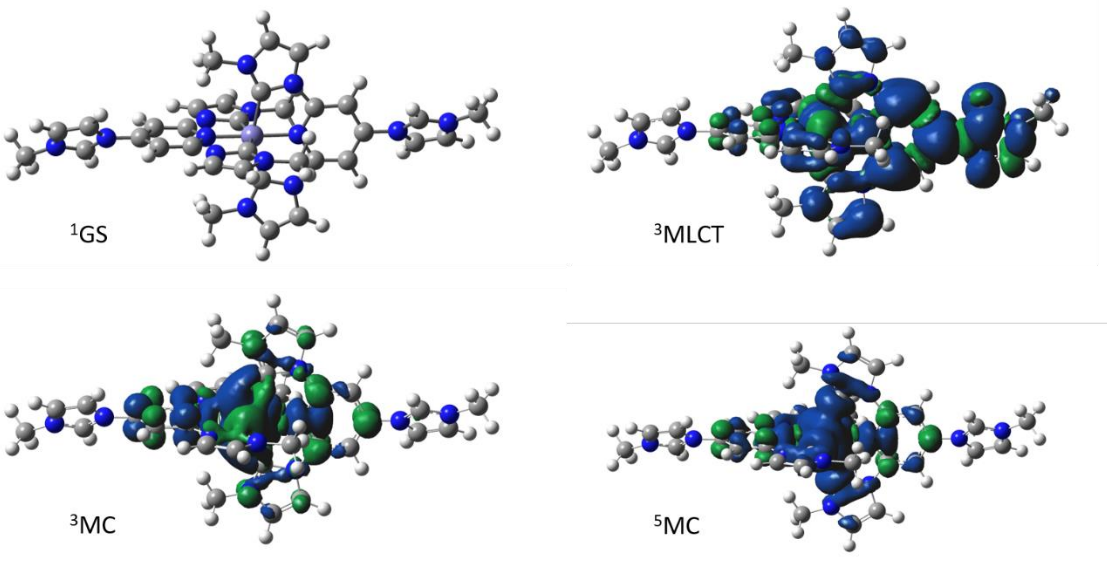

Figure S24. Calculated spin density contour plots of selected states of $[Fe(miHpbmi)_2](PF_6)_4$.

Table S6. Mulliken spin density on Fe, average Fe-C/N bond length and state energy relative to the ^{1}GS state of the optimized $[Fe(miHpbmi)_2](PF_6)_4$ states shown in Figure S24.

State	Spin density Fe	Fe-C/N bond length (Å)	Energy (eV)
^{1}GS	0	1.96	0
^{3}MLCT	1.03	1.98	1.72
^{3}MC	2.09	2.09	1.32
^{5}MC	3.74	2.23	1.69

Table S7. Single point energy at different optimized geometries for singlet, triplet and quintet surfaces. The potential energy surface landscape is visualized in the main manuscript, Figure 8a. The optimized ^{1}GS is put to 0 eV.

Energy (eV)	Singlet	Triplet	Quintet
^{1}GS	0.00	1.88	3.74
^{3}MLCT	0.24	1.72	3.31
^{3}MC	0.75	1.32	2.46
^{5}MC	1.90	1.99	1.69

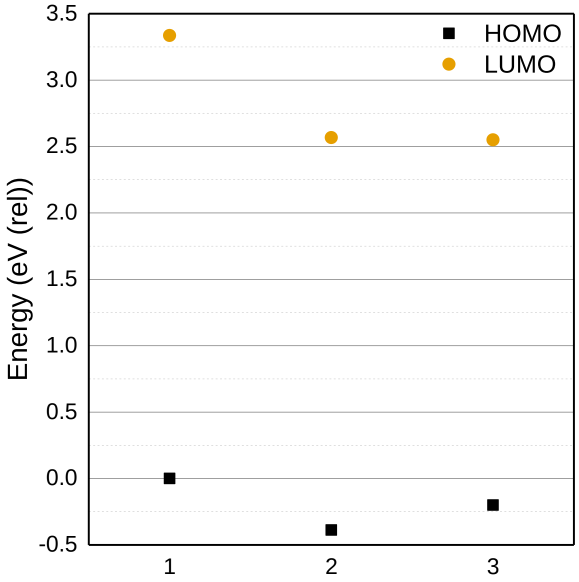

Figure S25. Relative energy of HOMO and LUMO orbitals of $[Fe(miHpbmi)_2](PF_6)_4$ (2) compared to $[Fe(pbmi)_2](PF_6)_2$ (1) and $[Fe(cpbmi)_2](PF_6)_2$ (3). The HOMO of $[Fe(pbmi)_2](PF_6)_2$ is put to 0 eV.

Table S8. The first 40 allowed singlet-singlet transitions from the ^{1}GS to higher singlet states of $[Fe(miHpbmi)_2](PF_6)_4$, calculated by TD-DFT. The nature of each transition is deemed by the involved Kohn-Sham molecular orbitals.

Excited state	Wavelength (nm)	Oscillator strength	Main molecular orbitals involved	Nature of transition
1	545.25	0.0005	180->181 (HOMO->LUMO)	MLCT
2	544.67	0.0007	180->182	MLCT
3	481.69	0.0001	178->182, 179->181	MLCT
4	474.65	0	178->181, 179->182	MLCT
5	442.79	0	180->183, 178->181, 179->182	MLCT

6	440.63	0.2287	178->182, 179->181, 180->184	MLCT
7	412.25	0.0013	179->187, 179->183, 179->189	MC/MLCT
8	407.42	0.0007	178->187, 178->189, 178->183	MC/MLCT
9	406.03	0.2968	180->184, 179->181, 178->182	MLCT
10	389.62	0.0077	178->183	MLCT
11	386.13	0.0075	179->183, 179->187, 179->189	MC/MLCT
12	373.23	0.0043	179->184	MLCT
13	372.69	0.004	178->184	MLCT
14	363.35	0	180->183, 179->182, 178->181	MLCT
15	348.91	0.0001	180->187, 180->189	MC
16	347.27	0	180->193	MC
17	335.14	0.0052	177->181	LC
18	334.55	0.0054	177->182	LC
19	328.10	0.0141	180->185	MLCT
20	327.02	0.0138	180->186	MLCT
21	324.62	0.0019	175->181, 176->182	LC
22	321.06	0	176->182, 175->181	LC
23	320.84	0	176->181, 175->182	LC
24	319.35	0	175->182, 176->181	LC
25	299.08	0.0003	178->185	MLCT
26	298.62	0.0046	179->186	MLCT
27	298.59	0.0001	179->185	MLCT
28	297.03	0	178->186	MLCT
29	291.51	0.0261	179->193, 174->181	MC/LC

30	291.51	0.0261	174->182, 178->193	MC/LC
31	287.76	0.0105	174->181, 179->193, 176->183	MC/LC
32	287.42	0.1797	177->183	LC
33	287.25	0.0102	174->182, 178->193	MC/LC
34	283.07	0	177->184	LC
35	282.59	0.0011	176->183	LC
36	282.40	0.0012	175->183	LC
37	279.03	0.0007	173->181, 176->184	LC
38	278.73	0.0003	175->184, 173->182	LC
39	278.41	0.0028	176->184, 173->181, 180->188	LC/MLCT
40	278.13	0.0031	173->182, 175->184	LC

S8. References

S1. Sheldrick, G. M. Crystal structure refinement with *SHELXL*. *Acta Cryst.* **2015**, *C71*, 3-8. DOI: 10.1107/S2053229614024218.

S2. Sheldrick, G. M. A Short History of *SHELXL Acta Cryst.* **2008**, *A64*, 112-122. DOI: 10.1107/S0108767307043930.

S3. CrysAlis PRO. Agilent Technologies **2013**. www.agilent.com/cs/library/usermanuals/Public/CrysAlis_Pro_User_Manual.pdf.

S4. Dolomanov, O. V.; Bourhis, L. J.; Gildea, R. J.; Howard, J. A. K.; Puschmann, H. *OLEX2*: a complete structure solution, refinement and analysis program *J. Appl. Cryst.* **2009**, *42*, 339-341. DOI: 10.1107/S0021889808042726.

S5. Müller, C.; Pascher, T.; Eriksson, A.; Chabera, P.; Uhlig, J. KiMoPack: A python Package for Kinetic Modeling of the Chemical Mechanism. *J. Phys. Chem. A* **2022**, *126*, 4087-4099. DOI: 10.1021/acs.jpca.2c00907.

S6. Wohlfarth, C. Viscosity of the mixture (1) acetonitrile; (2) tetrahydrofuran, part of Volume 25. Viscosity of Pure Organic Liquids and Binary Liquid Mixtures. Supplement to IV/18 of Landolt-Börnstein Group IV Physical Chemistry, **2009**. DOI: 10.1007/978-3-540-75486-2_704.

S7. Frisch, M. J.; Trucks, G. W; Schlegel, H. B.; Scuseria, G.E; Robb, M. A.; Cheeseman, J. R.; Scalmani, G.; Barone, V.; Mennucci, B.; Petersson, G. A.; Nakatsuji, H.; Caricato, M.; Li, X.; Hratchian, H. P.; Izmaylov, A. F.; Bloino, J.; Zheng, G.; Sonnenberg, J. L.; Hada, M.; Ehara, M.; Toyota, K.; Fukuda, R.; Hasegawa, J.; Ishida, M.; Nakajima, T.; Honda, Y.; Kitao, O.; Nakai, H.; Vreven, T.; Montgomery, J. A., Jr.; Peralta, J. E.; Ogliaro, F.; Bearpark, M.; Heyd, J. J.; Brothers, E.; Kudin, K. N.; Staroverov, V. N.; Kobayashi, R.; Normand, J.; Raghavachari, K.; Rendell, A.; Burant, J. C.; Iyengar, S. S.; Tomasi, J.; Cossi, M.; Rega, N.; Millam, N. J.; Klene, M.; Knox, J. E.; Cross, J. B.; Bakken, V.; Adamo, C.; Jaramillo, J.; Gomperts, R.; Stratmann, R. E.; Yazyev, O.; Austin, A. J.; Cammi, R.; Pomelli, C.; Ochterski, J. W.; Martin, R.L.; Morokuma, K.; Zakrzewski, V. G.; Voth, G. A.; Salvador, P.; Dannenberg, J. J.; Dapprich, S.; Daniels, A.D.; Farkas, Ö.; Foresman, J. B.; Ortiz, J. V.; Cioslowski, J.; Fox, D. J. Gaussian 09, Revision **C.01**. Gaussian, Inc., Wallingford CT, **2009**. https://gaussian.com/g09_c01/.

S8. Reiher, M.; Salomon, O.; B. Artur Hess. Reparameterization of hybrid functionals based on energy differences of states of different multiplicity *Theor. Chem. Accounts Theory, Comput. Model. (Theoretica Chim. Acta)* **2001**, *107 (1)*, 48–55. DOI: 0.1007/s00214-001-0300-3.

S9. Krishnan, R.; Binkley, J. S.; Seeger, R.; Pople, J. A. Self-consistent molecular orbital methods. XX. A basis set for correlated wave functions**.** *J. Chem. Phys*. **1980**, *72*, 650–654. DOI: 10.1063/1.438955.

S10. McLean, A. D.; Chandler, G. S. Contracted Gaussian basis sets for molecular calculations. I. Second row atoms, Z=11-18. *J. Chem. Phys.* **1980**, *72*, 5639–5648. DOI: 10.1063/1.438980.
